# Disrupting integrator complex subunit INTS6 causes neurodevelopmental disorders and impairs neurogenesis and synapse development

**DOI:** 10.1172/JCI191729

**Published:** 2025-09-18

**Authors:** Xiaoxia Peng, Xiangbin Jia, Hanying Wang, Jingjing Chen, Xiaolei Zhang, Senwei Tan, Xinyu Duan, Can Qiu, Mengyuan Hu, Haiyan Hou, Ilaria Parenti, Alma Kuechler, Frank J. Kaiser, Alicia Renck, Raymond Caylor, Cindy Skinner, Joseph Peeden, Benjamin Cogne, Bertrand Isidor, Sandra Mercier, Gael Nicolas, Anne-Marie Guerrot, Flavio Faletra, Luciana Musante, Lior Cohen, Gaber Bergant, Goran Čuturilo, Borut Peterlin, Andrea Seeley, Kristine Bachman, Julian A. Martinez-Agosto, Conny van Ravenswaaij-Arts, Dennis Bos, Katherine H. Kim, Tobias Bartolomaeus, Zelia Schmederer, Rami Abou Jamra, Erfan Aref-Eshghi, Wenjing Zhao, Yongyi Zou, Zhengmao Hu, Qian Pan, Faxiang Li, Guodong Chen, Jiada Li, Zhangxue Hu, Kun Xia, Jieqiong Tan, Hui Guo

**Affiliations:** 1 Hunan Key Laboratory of Medical Genetics, Hunan Key Laboratory of Animal Models for Human Diseases, MOE Key Lab of Rare Pediatric Diseases, Center for Medical Genetics, School of Life Sciences, Central South University, Changsha, Hunan, China.; 2 Furong Laboratory, Changsha, Hunan, China.; 3 Department of Pediatrics, Daping Hospital, Army Medical University, Chongqing, China.; 4 Institute of Human Genetics, University Hospital Essen, University of Duisburg-Essen, Essen, Germany.; 5 Center for Rare Diseases (Essener Zentrum für Seltene Erkrankungen), University Hospital Essen, Essen, Germany.; 6 Dricoll Children’s Hospital, Corpus Christi, Texas, USA.; 7 Greenwood Genetic Center, Greenwood, South Carolina, USA.; 8 East Tennessee Children’s Hospital, The University of Tennessee Department of Medicine, Knoxville, Tennessee, USA.; 9 Nantes Université, CHU de Nantes, CNRS, INSERM, l’institut du thorax, F-44000 Nantes, France.; 10 Nantes Université, CHU de Nantes, Service de Génétique médicale, F-44000 Nantes, France.; 11 Université Rouen Normandie, Normandie Université, INSERM U1245 and CHU Rouen, Department of Genetics and reference center for developmental abnormalities, F-76000 Rouen, France.; 12 Institute of Medical Genetics, Azienda Sanitaria Universitaria Friuli Centrale, Udine, Italy.; 13 Department of Medicine, University of Udine, Udine, Italy.; 14 Institute for Maternal and Child Health, IRCCS “Burlo Garofolo,” Trieste, Italy.; 15 Genetics Unit, Barzilai University Medical Center, Ashkelon, Israel.; 16 Faculty of Health Sciences, Ben-Gurion University of the Negev, Be’er Sheva, Israel.; 17 Centre for Mendelian Genomics, Clinical Institute of Genomic Medicine, UMC Ljubljana, Ljubljana, Slovenia.; 18 Faculty of Medicine, University of Belgrade, Belgrade, Serbia.; 19 University Children’s Hospital, Belgrade, Serbia.; 20 Medical Genetics, Geisinger Medical Center, Danville, Pennsylvania, USA.; 21 Department of Human Genetics, Division of Medical Genetics, Department of Psychiatry, Semel Institute for Neuroscience and Human Behavior, Department of Pediatrics, David Geffen School of Medicine at UCLA, Los Angeles, California, USA.; 22 University of Groningen, University Medical Centre Groningen, Department of Genetics, Groningen, Netherlands.; 23 Division of Genetics, Genomics, and Metabolism, Ann & Lurie Children’s Hospital of Chicago, Department of Pediatrics, Northwestern University, Feinberg School of Medicine, Chicago, Illinois, USA.; 24 Institute of Human Genetics, University of Leipzig Medical Center, Leipzig, Germany.; 25 Medical Genetics Center, Munich, Germany.; 26 GeneDx, LLC, Gaithersburg, Maryland, USA.; 27 Department of Medical Genetics, NHC Key Laboratory of Healthy Birth and Birth Defect Prevention in Western China, The First People’s Hospital of Yunnan Province, The Affiliated Hospital of Kunming University of Science and Technology, Kunming, Yunnan, China.; 28 School of Medicine, Kunming University of Science and Technology, Kunming, Yunnan, China.; 29 Department of Medical Genetics, Jiangxi Maternal and Child Health Hospital, Nanchang, Jiangxi, China.; 30 MOE Key Lab of Rare Pediatric Diseases, School of Basic Medicine, Hengyang Medical College, University of South China, Hengyang, Hunan, China.; 31 NHC Key Laboratory of Birth Defect for Research and Prevention, Hunan Provincial Maternal and Child Health Care Hospital, Changsha, Hunan, China.

**Keywords:** Genetics, Neuroscience, Genetic variation

## Abstract

The Integrator complex plays essential roles in RNA polymerase II (RNAPII) transcription termination and RNA processing. Here, we identify INTS6, a subunit of the Integrator complex, as a novel gene associated with neurodevelopmental disorders (NDDs). Through analysis of large NDD cohorts and international collaborations, we identified 23 families harboring monoallelic likely gene-disruptive or de novo missense variants in INTS6. Phenotypic characterization revealed shared features, including language and motor delays, autism, intellectual disability, and sleep disturbances. Using a nervous-system conditional KO (cKO) mouse model, we show that Ints6 deficiency disrupts early neurogenesis, cortical lamination, and synaptic development. Ints6 cKO mice had a thickened ventricular zone/subventricular zone, thinning of the cortical plate, reduced neuronal differentiation, and increased apoptosis in cortical layer 6. Behavioral assessments of heterozygous mice revealed deficits in social novelty preference, spatial memory, and hyperactivity, mirroring phenotypes observed in individuals with INTS6 variants. Molecular analyses further revealed that INTS6 deficiency alters RNAPII dynamics, disrupts transcriptional regulation, and impairs synaptic gene expression. Treatment with a CDK9 inhibitor (CDK9i) reduced RNAPII phosphorylation, thereby limiting its binding to target genes. Notably, CDK9i reversed neurosphere overproliferation and rescued the abnormal dendritic spine phenotype caused by Ints6 deficiency. This work advances understanding of INTS-related NDD pathogenesis and highlights potential therapeutic targets for intervention.

## Abstract 2

Dysfunction of INTS6 leads to a newly neurodevelopmental disorder in humans and is associated with impaired cortical development, disrupted synaptic maturation, and behavioral deficits in mice.

## Graphical abstract

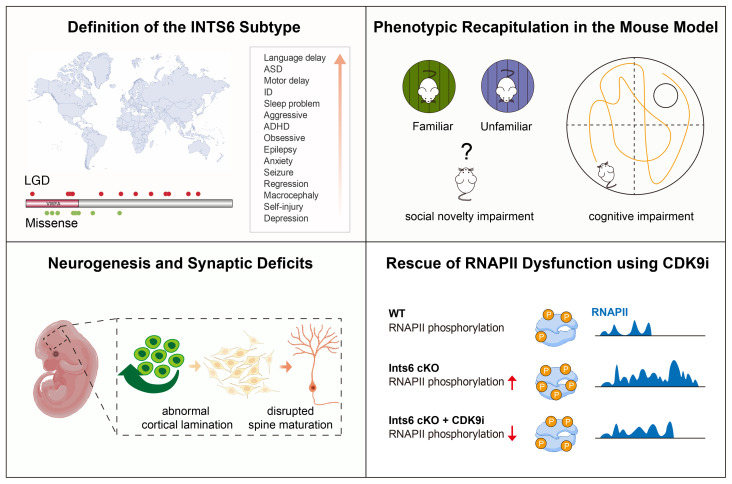

## Introduction

Neurodevelopmental disorders (NDDs) encompass a heterogeneous group of conditions characterized by impairments in cognitive, social, and motor functions, typically manifesting early in childhood. These disorders frequently exhibit a broad spectrum of overlapping clinical features (1). The etiology of NDDs is highly complex and multifactorial, with genetic mutations increasingly recognized as substantial contributors. Recent large-scale genomic studies have identified rare de novo mutations associated with NDDs, such as autism spectrum disorder (ASD) and intellectual disability, particularly in genes involved in transcriptional regulation, chromatin remodeling, and RNA processing (2–4). Despite advances in genomic research, the genetic landscape of NDDs remains only partially understood, due to the broad range of implicated genes and variability in clinical presentations.

The Integrator (INTS) complex is a pivotal multiprotein assembly involved in gene expression regulation, playing essential roles in RNA polymerase II (RNAPII) transcription termination and the processing of small nuclear RNAs (snRNAs) with de novo variants recently identified as a cause of neurodevelopmental syndromes (5–8). Comprising 15 core subunits (INTS1–INTS15), the INTS complex mediates RNA processing, ensuring transcriptional fidelity and proper mRNA maturation. These functions are particularly crucial in the central nervous system, where precise gene regulation supports neural development, differentiation, and synaptic plasticity. Although each subunit of the INTS complex contributes uniquely to these processes, the molecular roles of many subunits remain incompletely characterized. While disruptions in transcriptional and post-transcriptional regulatory mechanisms have been linked to NDDs, the specific involvement of the INTS complex in disease pathogenesis has only recently been recognized. Mutations in certain subunits, such as *INTS1*, *INTS8*, and *INTS11*, have been associated with NDDs (9, 10), suggesting that dysfunction of the INTS complex may underlie these conditions. However, for most INTS subunits, the connection with NDDs remains largely unexplored.

In this study, we analyzed genomic data from large NDD cohorts to investigate de novo mutations in all *INTS1*–*15* subunits and their potential associations with NDDs. We observed significant enrichment of de novo variants in *INTS6*, implicating its dysfunction in NDD pathogenesis. To further explore this relationship, we conducted an international, multicenter collaboration to assemble a cohort of cases with *INTS6* variants. These individuals exhibited common NDD phenotypes, including language, social, cognitive, and sleep impairments. Using a conditional KO (cKO) mouse model, we demonstrated that loss of *Ints6* disrupts early neurogenesis and RNAPII function. Mice with *Ints6* haploinsufficiency exhibited substantial social and cognitive deficits, along with impaired synaptic development. These findings provide strong evidence linking INTS6 dysfunction to neurodevelopmental outcomes, advancing our understanding of INTS-related pathogenesis in NDDs.

## Results

### Expression patterns and genetic associations of INTS genes with NDDs.

**Figure 1:**
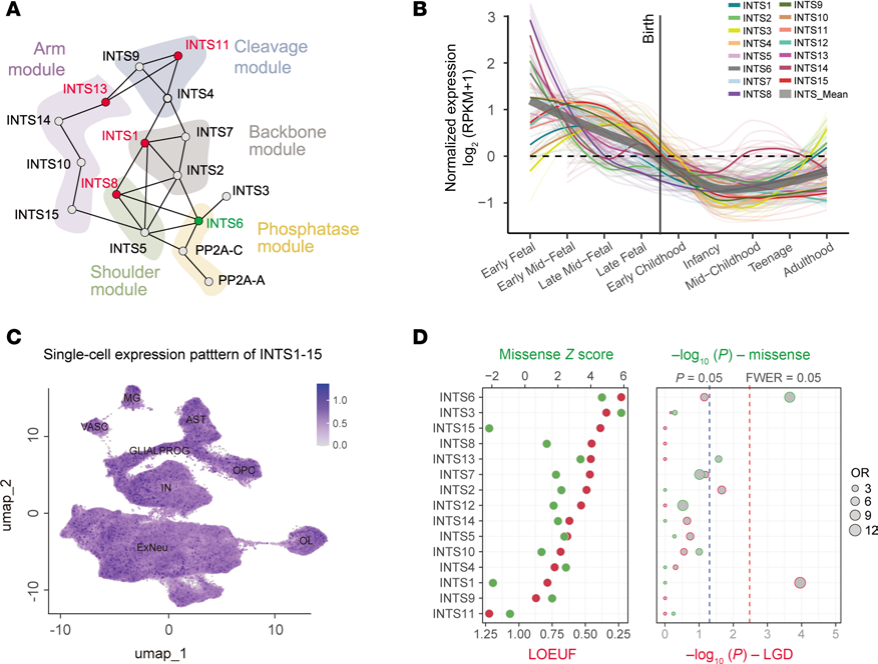
Expression patterns of *INTS1–15* in the human brain and enrichment of de novo variants in NDD cohorts. (**A**) Schematic representation of binary interactions within the INTS-PP2A complex, adapted from Offley et al. (62). Genes highlighted in red represent well-known NDD genes. (**B**) Normalized expression levels of *INTS1*–*15* genes across various developmental stages, including fetal stages, birth, infancy, childhood, teenage, and adulthood. Expression data are displayed as log_2_^(RPKM+1)^ values, with color coding highlighting gene-specific trends over time. (**C**) UMAP plot of single-cell RNA-Seq data showing the average expression patterns of INTS1–15 genes across distinct cell types, including microglia, astrocytes (AST), oligodendrocyte progenitor cells (OPC), excitatory neurons (ExNeu), inhibitory neurons (IN), vascular cells, and glial progenitors (GLIALPROG). (**D**) Left: Gene constraint metrics, including loss-of-function observed/expected upper bound fraction (LOEUF) scores and missense *Z* scores for loss-of-function (LGD) and missense variants, respectively, reflecting the genetic tolerance of INTS genes. Right: Enrichment analysis showing the significance of de novo LGD or missense variants in *INTS1*–*15* among NDD cohorts, compared with expected random occurrences. FWER,family-wise error rate.

The INTS complex comprises 15 proteins encoded by the *INTS1*–*15* genes. Functional interaction between the INTS complex and protein phosphatase 2A (PP2A) coordinates transcriptional and post-transcriptional regulation with phosphatase activity (Figure 1A). *INTS* genes are broadly expressed in the human brain and other tissues, as shown in the Genotype-Tissue Expression data set (Supplemental Figure 1; supplemental material available online with this article; https://doi.org/10.1172/JCI191729DS1). To investigate the temporal-spatial and single-cell expression patterns of INTS genes in the developing human brain, we analyzed the bulk RNA-Seq data from the BrainSpan project (https://www.brainspan.org/static/download.html) and a single-cell transcriptome data set (11). Our analysis revealed that the overall mean expression patterns of *INTS* genes in the BrainSpan data set are highly expressed during early brain development and progressively decrease after birth (Figure 1B). These findings indicate the INTS complex plays a vital role in brain development. Furthermore, single-cell transcriptome analysis demonstrated that *INTS1*–*15* are broadly expressed across various cell types in the developing human brain, further highlighting their potential importance in neural development (Figure 1C).

To examine the association between monoallelic variants in *INTS* genes and NDDs, we analyzed de novo variant data from 63,408 probands with NDDs, across multiple unpublished and published whole-exome and whole-genome sequencing data sets, including the Simons Foundation Powering Autism Research for Knowledge (SPARK) cohort (12), the Deciphering Developmental Disorders cohort (2), the Simons Simplex Collection (SSC) cohort (13), the Autism Sequencing Consortium cohort (14), and the MSSNG cohort (15) (see Methods, Supplemental Table 1). Using a Poisson distribution model (16), we compared the mutation rates of *INTS1*–*15* in the NDD cohorts with the expected random occurrence rates. Our analysis identified a significant enrichment of de novo likely gene-disruptive (LGD) variants in *INTS1* and de novo missense variants in *INTS6* among individuals with NDDs (adjusted *P* < 0.05, Bonferroni correction) (Figure 1D and Supplemental Table 2). Although biallelic variants in *INTS1* have been linked to NDDs (10), our findings suggest monoallelic loss-of-function variants in *INTS1* may also contribute to NDD risk. Notably, to our knowledge, *INTS6* has not been previously associated with NDDs. Given its novel genetic implication and the constraint observed for both missense and LGD variants (Figure 1D), we focused our subsequent analysis on understanding the role of *INTS6* in NDDs.

### Recruitment of a cohort of individuals with INTS6 variants.

**Figure 2:**
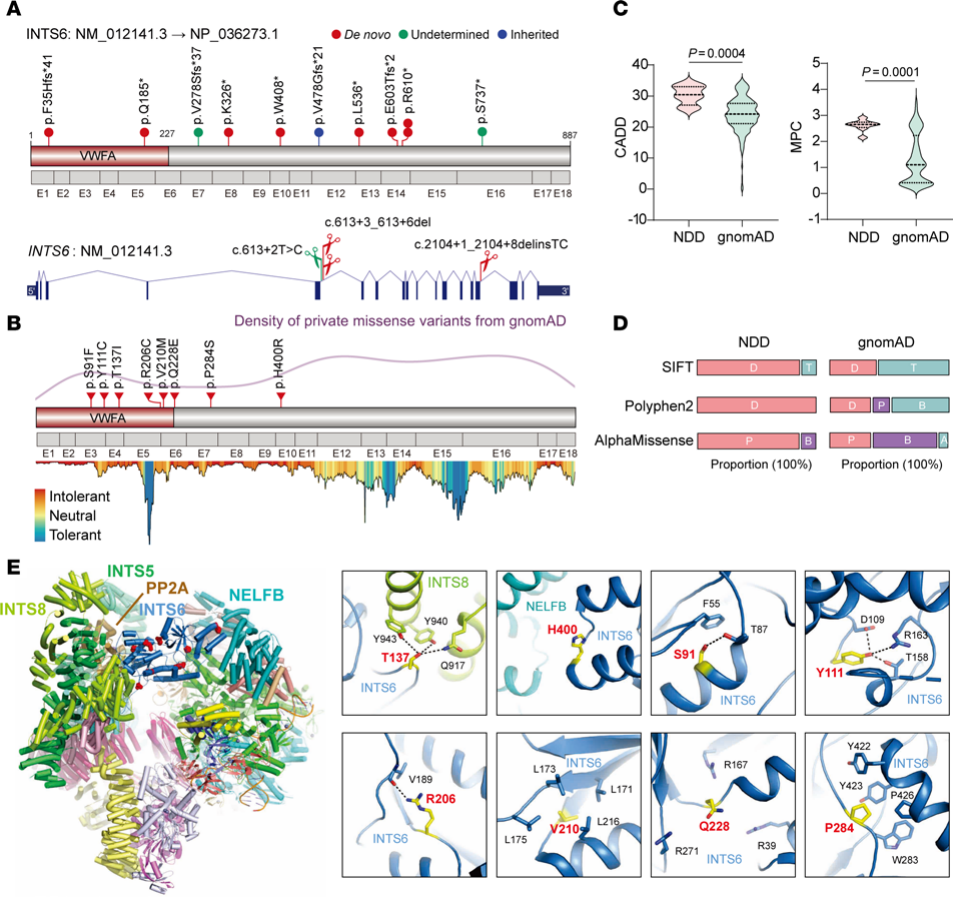
Monoallelic variants in *INTS6* lead to a new NDD syndrome. (**A**) The distributions of nonsense, frameshift, and splicing variants in *INTS*6 identified in NDDs are shown in a protein model and gene model, respectively. (**B**) The distribution of missense variants in *INTS6* identified in NDDs is shown in a protein model. Protein tolerance landscape for missense variants in *INTS6* was visualized via MetaDome20. All variants in INTS6 are predicted to be “intolerant” for aa substitutions. The density plot of ultrarare missense variants in gnomAD is shown. (**C**) Comparison of the distribution of combined annotation-dependent depletion (CADD) and MPC scores between de novo missense variants in NDDs and ultrarare missense variants in gnomAD database. Data are reported as mean ± SEM. *P* values were determined from a 2-tailed, unpaired Mann-Whitney test. (**D**) Comparison of SIFT, PolyPhen-2, and AlphaMissense prediction between de novo missense variants in NDDs and ultrarare missense variants in the gnomAD database. SIFT: D (deleterious), T (tolerated); PolyPhen-2: D (probably damaging), P (possibly damaging), B (benign); AlphaMissense: P (likely pathogenic), B (likely benign), A (ambiguous). (**E**) Left: Ribbon diagram of the INTS-PP2A complex bound to paused Pol II (PDB:7PKS). The disease-associated protein INTS6 and its interacting proteins are labeled. Right: Close-up view of NDD-related variants on INTS6 (red spheres), highlighting the importance of these residues in mediating protein-protein interactions or maintaining the structural integrity of INTS6.

To investigate whether *INTS6* variants lead to a novel NDD, we conducted a multicenter international collaboration facilitated by GeneMatcher (17). Through this effort, we identified 21 monoallelic variants within GeneMatcher *INTS6* in 24 affected individuals from 23 families (Supplemental Table 3). Among these variants, 13 were LGD variants, comprising 6 nonsense, 4 frameshift, and 3 putative splicing variants, identified in 15 families (Figure 2A). Notably, 2 independent families carried recurrent variants: p.R610* and c.613+3_613+6del. Functional analysis using a minigene assay revealed that the putative splice-site variant c.613+3_613+6del leads to exon 5 skipping (Supplemental Figure 2A), whereas the canonical splice-site variant c.2104+1_2104+8delinsTC results in exon 15 skipping (Supplemental Figure 2B). In addition to LGD variants, we identified 8 de novo missense variants in 9 affected individuals from 8 families, including an identical twin pair (p.S91F) (Figure 2B). All missense variants were located in the N-terminal region of *INTS6*, which is more conserved than the C-terminal, as evidenced by MetaDome analysis and a lower, ultra-rare missense variant density in the Genome Aggregation Database (gnomAD) cohort (18) (Figure 2B). Five of the 8 missense variants were located within the VWFA domain, which mediates interactions between the INTS complex and specific genes or transcriptional machinery. These de novo missense variants identified in affected individuals had higher combined annotation-dependent depletion (CADD) scores (19) and Missense badness, PolyPhen-2, and Constraint (MPC) scores (20) compared with ultrarare missense variants in the gnomAD cohort (Figure 2C). All but 1 missense variant were predicted to be damaging or probably damaging by the sorting intolerant from tolerant (SIFT) (21), Polymorphism Phenotyping v2 (PolyPhen-2) (22), and AlphaMissense (23) (Figure 2D) algorithms. These findings support the pathogenic potential of both LGD and missense variants in *INTS6*.

To further evaluate the potential pathogenic impact of disease-associated missense variants, we analyzed their effects from a structural perspective. The cryogenic electron microscopy structure of the INTS-PP2A complex bound to paused RNAPII revealed that INTS6 directly interacts with INTS8, INTS5, PP2A, and NELFB (Figure 2E). The affected amino acid residues can be categorized into 2 groups based on their structural locations and roles. Residues T137 and H400 are located at critical interfaces with INTS8 and NELFB, respectively. The side chain of T137 forms hydrogen bonds with Q917, Y940, and Y943 of INTS8, stabilizing the complex, and mutations such as T137I and H400R are likely to disrupt these interactions, impairing the binding of INTS6 to INTS8 and NELFB (Figure 2E).

Several other residues within INTS6 are crucial for maintaining its structural stability (Figure 2E): S91 is located near F55 and forms a hydrogen bond with the side chain of T87; the S91F mutation would eliminate this bond and introduce steric clashes with F55 due to the larger side chain. Similarly, Y111 and R206 form a hydrophilic network with D109, T158, R163, and the backbone of V189, and mutations Y111C and R206C would disrupt these contacts, destabilizing the structural fold. V210 resides within a hydrophobic pocket formed by L171, L173, L175, and L216, and the V210M mutation likely alters this hydrophobic environment. Q228 is positioned in a loop region without substantial residue contacts; however, nearby positively charged residues R39, R167, and R271 could form additional salt bridges with the negatively charged Q228E mutation, potentially altering local electrostatic interactions. Finally, P284 engages in hydrophobic interactions with W283, Y422, Y423, and P426, and replacing P284 with a hydrophilic serine (P284S) would disrupt these hydrophobic contacts, compromising protein folding (Figure 2E). In summary, the NDD-associated missense variants in *INTS6* might impact its function by either disrupting protein-protein interactions or destabilizing the structural integrity required for its activity, likely impairing the role of INTS6 within the INTS-PP2A complex and contributing to neurodevelopmental pathologies.

### INTS6 monoallelic variants are associated with core features of developmental delay and autism, with a potential male predominance.

**Figure 3:**
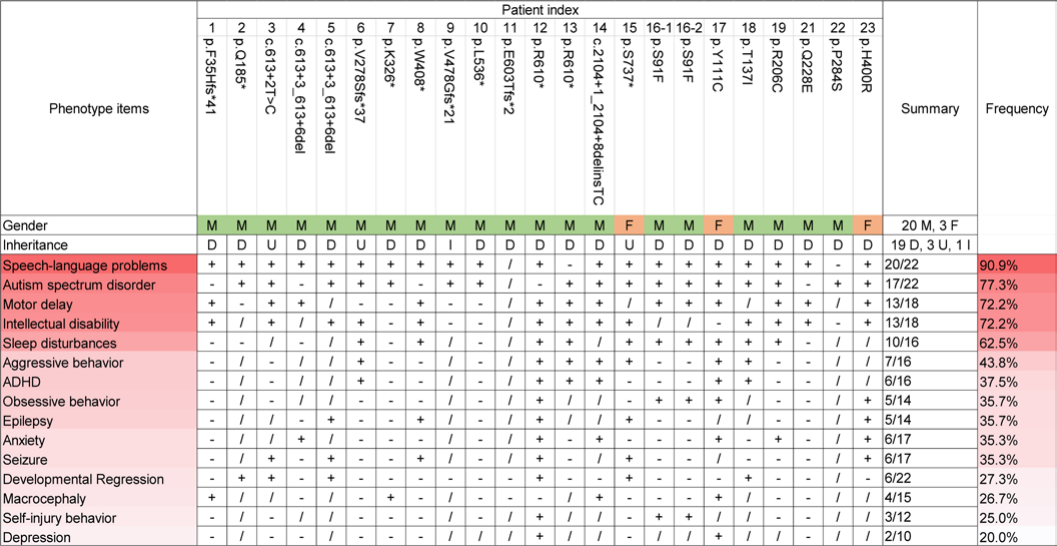
Phenotypic spectrum of individuals carrying *INTS6* variants. M, male; F, female; D, de novo; I, inherited; U, undetermined; +, present; –, absent; /, no data or undetermined.

To understand *INTS6*-related symptoms, we compiled detailed phenotypic data for 23 affected individuals carrying *INTS6* variants (Supplemental Table 4). All individuals exhibited neurodevelopmental concerns, with the most common features being language and motor delays, ASD, intellectual disability, and sleep disturbances (Figure 3). Specifically, speech and language problems were reported in 20 of 22 individuals who underwent language assessments. Among 22 individuals evaluated for ASD, 17 met the diagnostic criteria for ASD. Motor delays and intellectual disabilities were observed in 13 of 18 individuals. Sleep disturbances were noted in 10 of 16 individuals (Figure 3). Additionally, other neuropsychiatric and neurological issues were identified, including aggressive behavior (*n* = 7 of 16 individuals), attention-deficit hyperactivity disorder (ADHD) (*n* = 6 of 16), obsessive behavior (*n* = 5 of 14), epilepsy (*n* = 5 of 14), anxiety (*n* = 6 of 17), seizures (*n* = 6 of 17), developmental regression (*n* = 6 of 22), macrocephaly (*n* = 4 of 15), self-injurious behavior (*n* = 3 of 12), and depression (*n* = 2 of 10) (Figure 3). To investigate whether the phenotypes correlate with the type of variants, we performed unsupervised clustering analysis. The results showed no clear correlation between the clinical features and the type of variants (Supplemental Figure 3). In summary, these findings highlight a pattern of overlapping neurodevelopmental and neuropsychiatric features in individuals with *INTS6* variants, underscoring the shared phenotypic spectrum among affected individuals.

Interestingly, a notable gender bias was observed in the distribution of *INTS6* variants among affected individuals (Figure 3). Of the 15 individuals with de novo LGD variants or variants of unknown inheritance, 14 were male, with only 1 female identified. In the single family with inherited *INTS6* variants, the affected male proband inherited the variant from an unaffected mother. Additionally, among the 8 probands carrying de novo missense variants, 6 were male. These findings suggest a potential male predominance or female protective effect associated with *INTS6* variants in this disorder. To investigate whether this predominance is linked to differential expression of *INTS6* between male and female individuals, we performed a differential expression analysis using single-cell RNA-Seq data from human brain samples. However, no pronounced sex-related differences in *INTS6* expression were observed (Supplemental Figure 4, A and B).

### Ints6 deficiency interferes with neurogenesis and cortical lamination.

To investigate the role of INTS6 in early neurogenesis and cortical development, we generated a cKO model using CRISPR-Cas9 to insert loxP sites flanking exons 5 and 6 of the mouse *Ints6* gene. Mating with Nestin-Cre mice resulted in neural-specific *Ints6* deletion (24) (Supplemental Figure 5A). Genotypic analysis confirmed the expected Mendelian segregation (Supplemental Figure 5B), and mRNA analysis at embryonic day 16.5 (E16.5) revealed about a 50% reduction in *Ints6* expression in conditional heterozygous KO (cHET) mice compared with WT mice, demonstrating efficient gene targeting (Supplemental Figure 5C). Analysis of offspring genotypes adhered to Mendelian principles. Notably, *Ints6* cKO mice exhibited postnatal lethality within 7 days, whereas cHET mice grew comparably to WT control mice in terms of weight and overall health (Supplemental Figure 6A).

**Figure 4:**
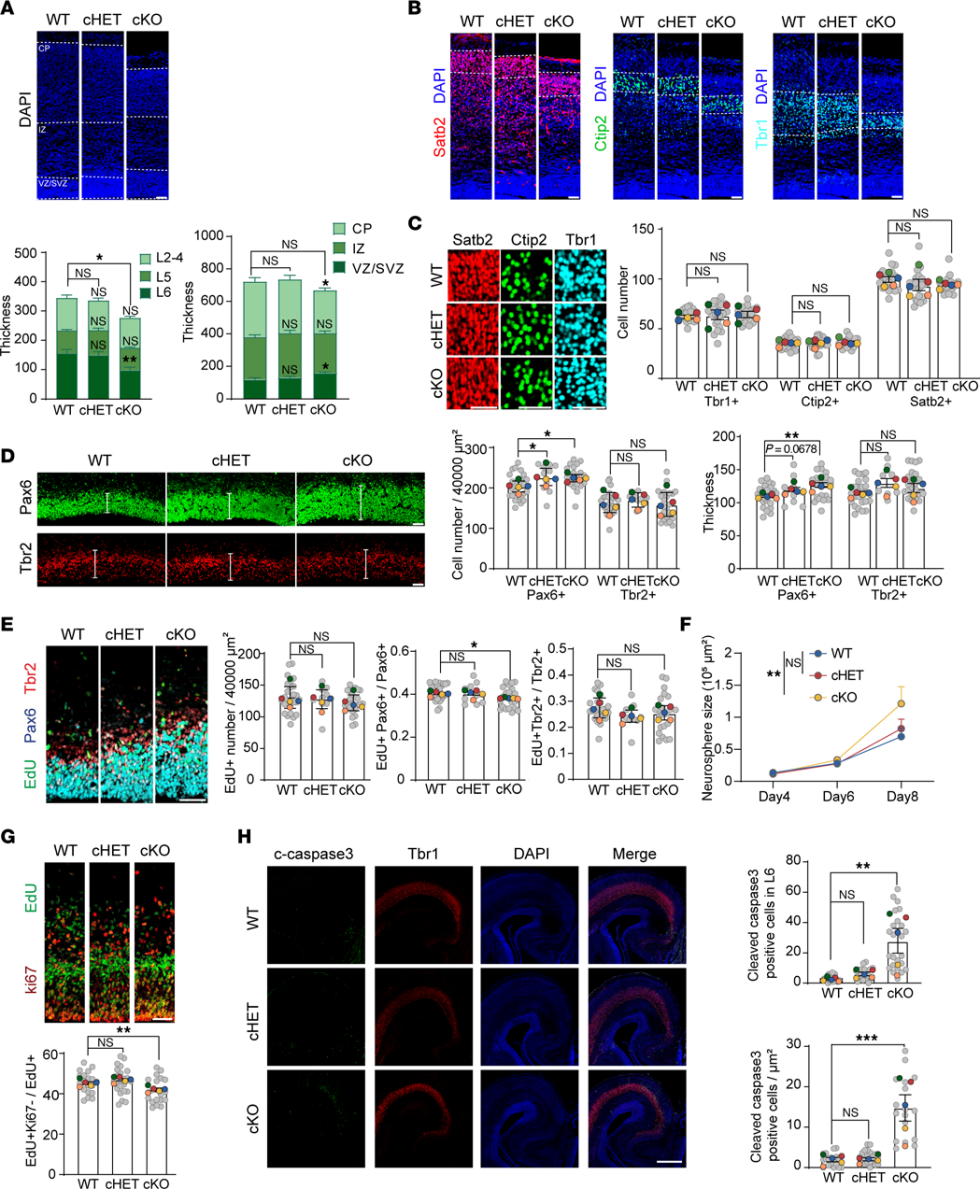
*Ints6* deficiency interferes with neurogenesis and cortical lamination. (**A**) DAPI staining of cortical sections from E18.5 WT, cHET, and cKO mice (*n* = 5 per group), with measurements of cortical thickness. White dashed lines indicate the VZ/SVZ, intermediate zone (IZ) and CP. (**B**) Immunofluorescence of E18.5 cortical sections stained for layer-specific markers: Satb2 (red) for layers II-IV, Ctip2 (green) for layer V, and Tbr1 (cyan) for layer VI. Cortical thickness was quantified across genotypes (*n* = 5). (**C**) Magnified views of cortical marker staining in **B** and statistical analysis of cell numbers, *n* = 5. (**D**) Pax6 (green) and Tbr2 (red) staining in the SVZ and VZ of E15.5 embryonic brains, comparing WT, cHET, and cKO (*n* = 5 per group). Quantification of cortical thickness and Pax6+ and Tbr2+ cells are shown. (**E**) Triple labeling with Pax6 (cyan), Tbr2 (red), and Edu (green) at 0.5 hours post-injection in E15.5 cortices of WT, cHET, and cKO mice, to evaluate proliferative dynamics (*n* = 5 per group). (**F**) Neurosphere growth curves (days 4, 6, and 8). *P* values were determined from 2-way ANOVA with Dunnett’s multiple comparisons test. (**G**) EdU (green) with 24-hour labeling and ki67 (red) staining in WT, cHET, and cKO cortex at E15.5, showing differentiative capacity. (**H**)Tbr1 (red) and cleaved-caspase-3 (green) staining in E18.5 embryonic brains (*n* = 5 per group). DAPI-stained nuclei are shown in blue. *P < 0.05, **P < 0.01, ***P < 0.001. Scale bar: 50 μm in (**A**–**G**), 100 μm in (**H**). Each biological replicate (mouse) is color-coded; gray dots show individual data point, and colored dots indicate the mean per mouse. (**A**–**E, G, and H**) Data are reported as mean ± SEM. *P* values were determined from 1-way ANOVA with Dunnett’s multiple comparisons test.

Given the importance of cortical structure in NDD, we examined the gross morphology of the cerebral cortex. Measurements of the anterior-posterior axis and anterior-dorsal lengths in cKO mice showed no significant differences compared with WT control mice, indicating that *Ints6* deficiency does not alter overall cortical dimensions (Supplemental Figure 6, B–D). In exploring the role of *Ints6* in cortical layer development, we focused on the ventricular zone/subventricular zone (VZ/SVZ), intermediate zone, and cortical plate (CP)—critical areas for neuronal generation, migration, and organization. DAPI staining and quantification of cell nuclei in E18.5 tissue revealed a thickened VZ/SVZ in the *Ints6* cKO model. Conversely, the CP exhibited significant thinning, indicating impaired neuronal migration or maturation. These changes were not observed in cHET mice (Figure 4A and Supplemental Figure 7A). These findings underscore the critical role of *Ints6* in proper cortical layer formation.

We further assessed cell identity in the CP using layer-specific neuronal markers. Satb2 predominantly marks upper-layer (L2–L4) neurons, Ctip2 identifies a subset of deep-layer (L5) neurons involved in subcortical projection pathways, and Tbr1 labels early-born, deep-layer (L6) neurons. Although the density of Tbr1^+^ neurons was comparable, the thickness of L6 was significantly reduced in cKO mice. In contrast, the thickness and numbers of Satb2^+^ and Ctip2^+^ neurons did not differ significantly among cKO, cHET, and WT mice (Figure 4, B and C, and Supplemental Figure 7B). At an earlier developmental stage (E15.5), the region marked by Pax6, indicative of active neural progenitors, was thicker in the *Ints6* cKO mice, whereas the adjacent SVZ marked by the Tbr2 region showed no change in thickness (Figure 4D and Supplemental Figure 7C).

The thickening of SVZ and the thinning of CP L6 suggest a divergence between increased progenitor proliferation and impaired neuron differentiation or survival. To investigate this, we analyzed cortical proliferation in E15.5 mice. Although the total number of EdU^+^ cells after a 30-minute EdU pulse was unchanged, EdU incorporation in Pax6^+^ progenitors was reduced in cKO mice (*P* = 0.056), indicating a shortened cell cycle and accelerated proliferation of Pax6-expressing neural stem cells. In contrast, the progenitor of Tbr2^+^ intermediate cells was unaffected (Figure 4E and Supplemental Figure 7D).

We performed neurosphere formation assays to further investigate the impact of *Ints6* deficiency on neurogenesis. We recorded and measured the size of neurospheres at 4, 6, and 8 days in vitro and found that the *Ints6* cKO neurospheres grew more rapidly than those of the WT, which is consistent with our immunofluorescence staining results (Figure 4F and Supplemental Figure 7E).

Differentiation deficits were evident in E15.5 mice, where 24-hour EdU labeling showed fewer cells exiting the cell cycle, reflecting impaired differentiation of neural stem cells into neurons (Figure 4G). However, cortical neuron migration appeared unaffected, as indicated by the unchanged distribution of EdU^+^ cells across VZ/SVZ, intermediate zone, and CP regions (Supplemental Figure 7F), as well as between CP layers marked by Satb2, Ctip2, and Tbr1 (Supplemental Figure 7G). At E18.5, a significant increase in apoptosis was observed in L6 neurons, as indicated by elevated cleaved caspase-3 levels (Figure 4H). These findings suggests that while *Ints6* knockout accelerates neural stem cell proliferation and thickens the SVZ, it simultaneously hinders differentiation and promotes apoptosis, leading to L6 thinning and disrupted cortical stratification.

### Ints6 deficiency disrupts PP2A-RNAPII function.

**Figure 5:**
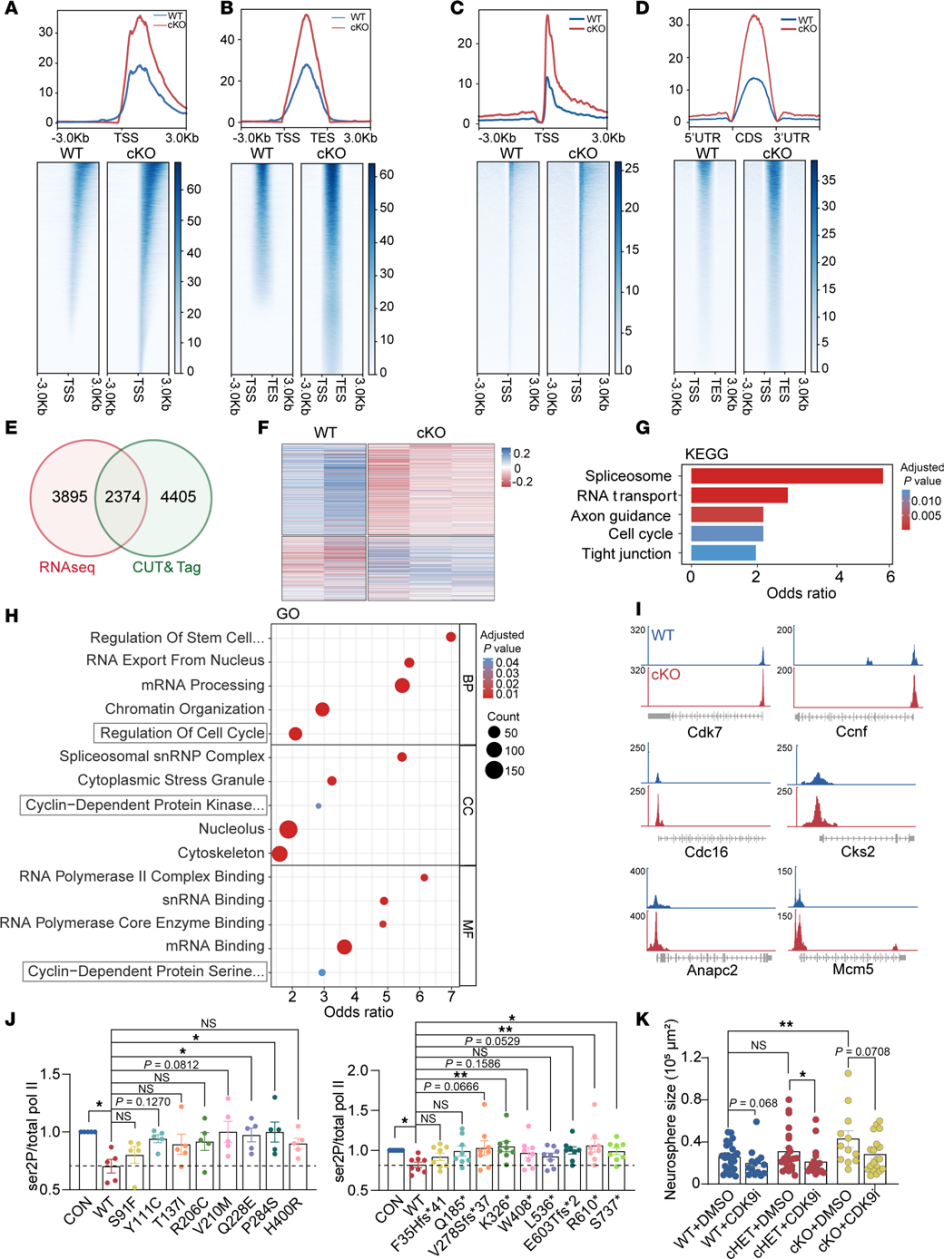
*Ints6* deficiency disrupts PP2A-RNAPII function. (**A**) Heatmap and spatial distribution of RNAPII binding at the TSS of genes analyzed by CUT&Tag in WT and cKO E15.5 mice. (**B**) Average distribution profile of RNAPII across gene regions, including TSSs and TESs. (**C**) Spatial distribution and heatmap representation of the distance of RNAPII binding around the TSS of RIP-Seq genes. (**D**) Average distribution profile of RNAPII across coding sequences (CDS) regions of RIP-Seq. The gradient of blue to white color (**A**–**D**) indicates high to low counts in the corresponding region. (**E**) A Venn diagram illustrating the overlap between DGEs (*P* < 0.05) identified in RNA-Seq and CUT&Tag data sets. (**F**) Heatmap analysis of the relative expression levels of 2,374 genes in the overlap of RNA-Seq and CUT&Tag. Upregulated genes are depicted in blue; downregulated genes are shown in red. (**G**) Bar graph depicting enriched KEGG pathways identified from the overlap data. (**H**) Bubble plot depicting enriched GO terms identified from the overlap data. (**I**) Browser tracks of CUT&Tag profiles for the genes related to the cell cycle at E15.5 days of embryonic development, comparing expression levels in WT and INTS6 cKO mice. (**J**) Western blot analysis of total RNAPII and Ser2P in HEK293T cells transfected with either WT, missense variants (*n* = 5), or LGD variants (*n* = 8). *P* values were determined from Friedman with Dunnett’s multiple comparisons test. (**K**) Statistical analysis of the effects of CDK9i on the growth of WT (*n* = 27 DMSO-treated; *n* = 14 CDK9i-treated); cHET (*n* = 23 DMSO-treated, *n* = 18 CDK9i-treated) and cKO (*n* = 12 DMSO-treated, *n* = 19 CDK9i-treated) neurosphere. *P* values were determined from a 2-tailed unpaired *t* test and Mann-Whitney test. *P < 0.05, **P < 0.01. CON, control. Data are reported as mean ± SEM.

INTS6 is a component of the INTS complex, a multi-subunit protein complex critical for RNA processing through its interaction with RNAPII (25). To examine the role of INTS6 in RNAPII function and neural development, we performed cleavage under targets and tagmentation (CUT&Tag) sequencing and RNA immunoprecipitation sequencing (RIP-Seq) using RNAPII antibody on E15.5 brain tissues from WT and cKO mice. CUT&Tag analysis revealed a significant enrichment of RNAPII near the transcription start site (TSS) and in distal promoter regions in *Ints6* cKO mice compared with WT mice (Figure 5A and Supplemental Table 5). Further examination of the genomic distribution of RNAPII binding peaks showed a notable increase in RNAPII occupancy across the entire gene body in *Ints6* cKO mice (Figure 5B). RIP-Seq analysis revealed a shift in RNA-binding distribution between WT and cKO brain tissues. In WT tissues, 35% of reads mapped to UTRs (17.6% in 5′ UTRs and 17.4% in 3′ UTRs) and 37.3% to intronic. In contrast, cKO tissues exhibited 37.8% in UTRs (18.6% in 5′ UTRs and 19.2% in 3′ UTRs) and 33% in intronic (Supplemental Figure 8A and Supplemental Table 6). Genome-wide profiling demonstrated elevated RNAPII accumulation near TSS regions in cKO tissues compared with WT (Figure 5C). Signal density analysis further revealed increased RNAPII occupancy at transcription end sites (TESs) and across genomic regions in cKO tissues (Figure 5D). Collectively, these findings suggest *Ints6* deficiency leads to enhanced RNAPII retention at key gene regulatory regions.

To explore transcriptomic changes, we conducted mRNA-Seq on E15.5 mouse brain tissue. Compared with the WT group, the *Ints6* cKO group displayed upregulation of the majority of differentially expressed genes (DEGs) (Supplemental Table 7). To refine these findings, we performed an overlap analysis between DEGs identified by RNA-Seq and genes with differential RNAPII binding identified by CUT&Tag. RNA-Seq revealed 6,269 DEGs, whereas CUT&Tag identified 6,779 genes with altered RNAPII binding, with 2,374 genes overlapping between the 2 data sets (Figure 5, E and F). Among the 2,374 DEGs, 791 were upregulated. Of these, RNAPII occupancy was increased in approximately 661 genes, and the remaining genes showed either decreased occupancy or no substantial change (Supplemental Figure 8, B and C). This finding indicates that RNAPII binding does not always directly correlate with mRNA expression levels, which is consistent with reports from previous studies (26). Gene Ontology (GO) and Kyoto Encyclopedia of Genes and Genomes (KEGG) pathway enrichment analyses of the overlapping genes revealed significant enrichment in RNA processing-related pathways and cell cycle–related pathways (Figure 5, G and H). The binding ability of RNAPII to key regulators of the cell cycle, including Cdk7 (27), Ccnf (28), Mcm5 (29), and Cks2 (30), as well as components of the anaphase-promoting complex/cyclosome, such as Cdc16 and Anapc2 (31, 32), were significantly increased in *Ints6* cKO mice (Figure 5I). Consistent with these results, the 3,031 overlapping genes between DEGs and RIP-Seq data were also significantly enriched in cell cycle–related pathways (Supplemental Figure 8, D–F). We extracted the total mRNA from the cortical tissue and confirmed by qPCR that the expression of cell cycle–related genes in the RIP-Seq analysis was downregulated (Supplemental Figure 8G). These findings are consistent with the disturbed cell cycle observed in neural progenitors of cKO mice.

To examine the impact of disorder-related *INTS6* variants on PP2A-RNAPII function, we overexpressed plasmids encoding the WT and the 8 disorder-associated missense and 9 disorder-associated LGD variants in HEK293T cells. Our results showed that WT INTS6 significantly suppressed RNAPII Ser2 phosphorylation (Ser2P), consistent with previous findings (33) (Figure 5J and Supplemental Figure 8H). Overall, all missense and LGD variants had higher Ser2P levels compared with WT, indicating a failure to suppress Ser2P. However, only 2 missense variants (V210M and Q228E) and 3 LGD variants (K326*, R610*, and S737*) reached statistical significance with the current number of replicates (Figure 5J and Supplemental Figure 8H). Because the inhibitor of CDK9 (CDK9i) could lead to a decrease in the phosphorylation of RNAPII, blocking its excessive binding to the target gene (Supplemental Figure 8I) (33), we then tested whether CDK9i could rescue INTS6-related pathogenesis via neurosphere formation assays. The result showed that CDK9i reversed the overproliferation of the *Ints6* cKO (Figure 5K and Supplemental Figure 8J). To further verify the impact of the variants on neural development, we constructed shRNA targeting *Ints6* to knockdown in the brain (Supplemental Figure 9A). The results of in utero electroporation showed that knockdown of *Ints6* led to a significant increase in Pax6^+^ cells, a finding consistent with that of previous results (Figure 4D). By coexpressing WT INTS6 or disorder-related variants plasmid (T137I and H400R), we found that the WT INTS6 could rescue the phenotype of increased Pax6^+^ cells, whereas the 2 variants located in the key region of the integrator could not (Supplemental Figure 9B). These findings indicate that disorder-related variants disrupt PP2A-RNAPII function, potentially contributing to neurogenesis defects as observed in the cKO mice.

### Ints6 haploinsufficiency leads to social and cognitive impairments in mice.

**Figure 6:**
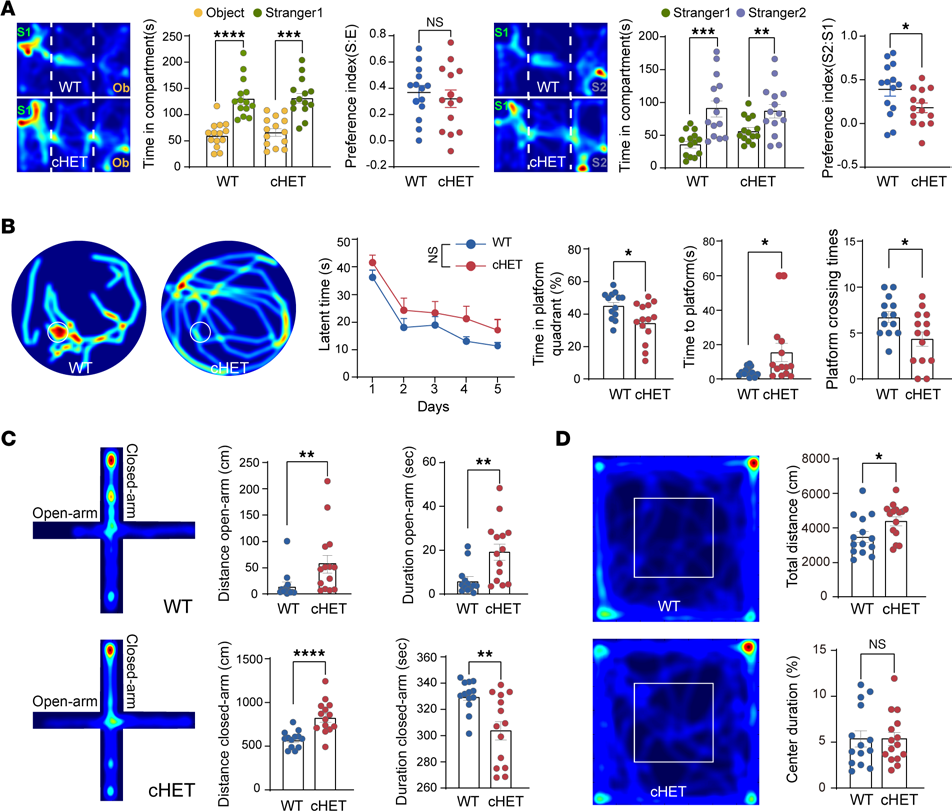
*Ints6* cHET mice lead to social and cognitive impairments. (**A**) Heatmaps depicting the movement of WT (*n* = 14) or cHET (*n* = 14) mice in a 3-chamber social interaction test. Preference scores were calculated as (S – E)/(S + E) for social versus empty interactions and (S2 – S1)/(S2 + S1) for stranger versus original mouse interactions. Data are reported as mean ± SEM. *P* values were determined from a 2-tailed unpaired *t* test. (**B**) Morris water maze test of spatial learning and memory in *Ints6* cHET mice. Latent time (s) during training trials, time in platform quadrant, and distance traveled are measured (*n* = 14). Data are reported as mean ± SEM. *P* values were determined from 2-way ANOVA with Bonferroni’s multiple comparisons test and a 2-tailed unpaired Mann-Whitney test. (**C**) Elevated cross-maze experiments were performed with WT (*n* = 13) and cHET (*n* = 14) mice to evaluate anxiety-related behaviors. The experiments statistically analyzed the movement distance and dwell time in both the open and closed arms of the maze. Data are reported as mean ± SEM. *P* values were determined from a 2-tailed unpaired Mann-Whitney test. (**D**) Path-tracking images from an open field test, showing movement patterns of WT (*n* = 14) and cHET (*n* = 15) mice. Bar graph representing the distance traveled and the time spent in the central area of the open field over 10 minutes. Data are reported as mean ± SEM. *P* values were determined from a 2-tailed unpaired *t* test. *P < 0.05, **P < 0.01, ***P < 0.001, ****P < 0.0001. snRNP, small nuclear ribonucleoprotein.

To further explore the role of *Ints6* in disease pathogenesis, we conducted a series of behavioral tests to evaluate common phenotypes associated with *INTS6* variants in humans. Given the lethality of cKO mice, our analyses focused on cHET mice to assess autism-related behaviors and cognition ability. Social behavior, a hallmark of autism, was evaluated using the 3-chamber social test. Although cHET mice did not show marked social interaction impairment, we observed significantly diminished social novelty preference in cHET mice compared with WT mice (Figure 6A), indicating social novelty impairment. Repetitive and stereotyped behaviors were assessed by monitoring rearing, grooming, and digging. No significant differences were observed in rearing or digging between *Ints6* cHET and WT mice, and grooming showed only a slight increase, indicating minimal impact on repetitive or stereotyped behaviors (Supplemental Figure 10A).

Cognitive abilities were assessed using Morris water maze, Y-maze, and novel object recognition tests. In the water maze, *Ints6* cHET mice showed reduced learning efficiency and impaired spatial memory retention, as evidenced by less time spent in the target quadrant during the testing phase; total swimming distance, however, remained unchanged (Figure 6B). In contrast, the Y-maze and novel object recognition tests revealed no significant differences between *Ints6* cHET and WT mice (Supplemental Figure 10, B and C).

Anxiety-related and hyperactive behaviors were assessed using the elevated plus maze, open field test, and bead-burying test. In the elevated plus maze, *Ints6* cHET mice had significantly increased movement time and distance within the open arms of the maze, alongside enhanced movement in the closed arms, suggesting that *Ints6* deficiency may contribute to hyperactivity rather than traditional anxiety-like behavior (Figure 6C). Similarly, in the open field test, the total distance traveled was significantly elevated, although time spent in the central area remained unchanged, further supporting a hyperactive phenotype (Figure 6D). In contrast, the bead-burying test revealed no significant differences in the number of beads buried within 30 minutes (Supplemental Figure 10D). Taken together, these findings suggest *Ints6* haploinsufficiency in mice is associated with reduced social novelty preference, impairments in spatial memory and learning, and hyperactivity.

### Ints6 deficiency interferes with synapse development.

**Figure 7:**
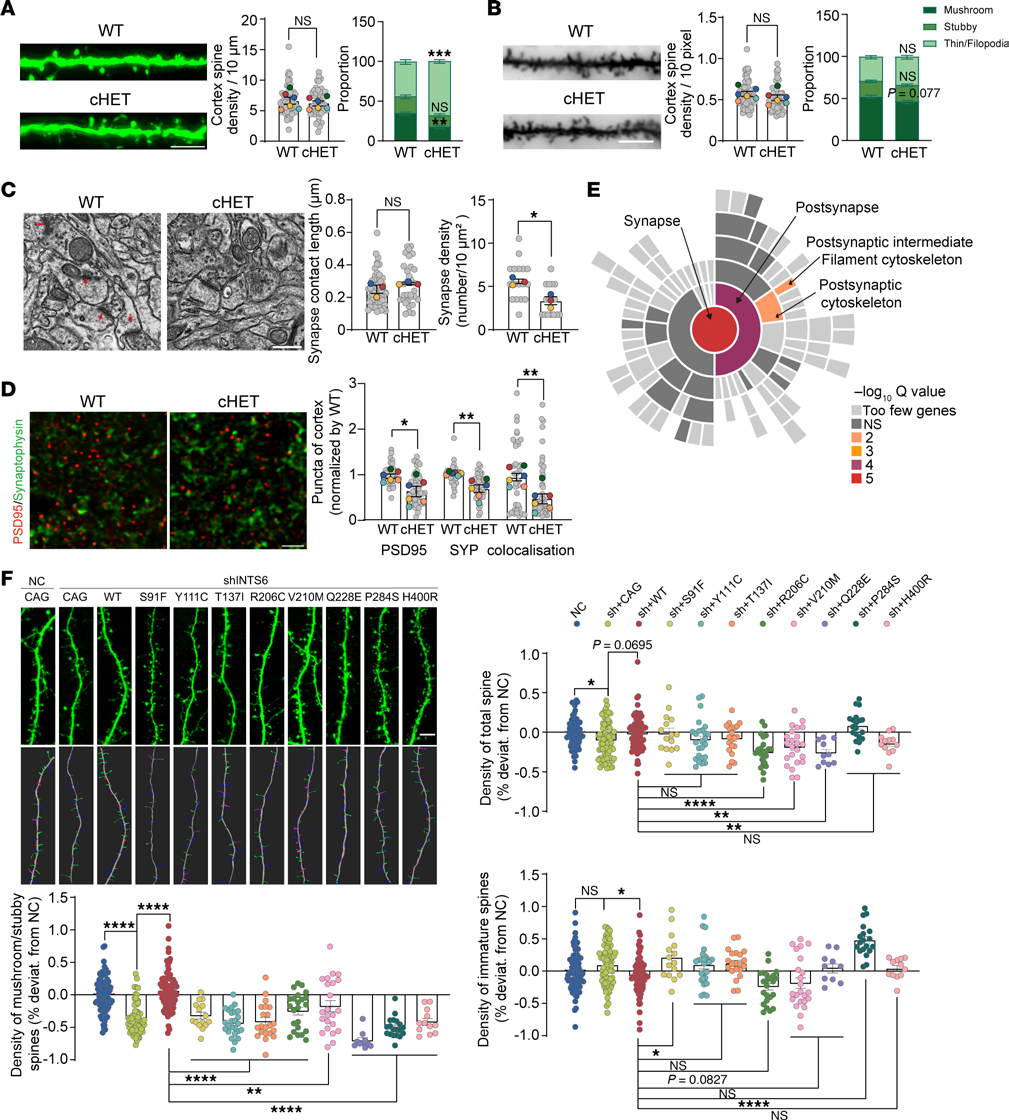
*Ints6* deficiency interferes synapse development. (**A**) Dendritic spine from 5-week-old mice harboring the Thy1-GFP transgene. Density of dendritic spine and the 3 different morphological spine types expressed as the number of spines normalized to 10 μm of dendritic length. Scale bar: 5 μm. (**B**) Golgi staining of dendritic spines in the cortex layer 2/3 neurons. Density of dendritic spine and the 3 different morphological spine types expressed as the number of spines normalized to 10 pixel of dendritic length. Scale bar: 100 pixels. *P* values were determined from a 2-tailed unpaired Mann-Whitney and *t* test. (**C**) Brain tissue slices from 7-week-old mice were imaged using electron microscopy to visualize synaptic structures. The red arrow indicates the dense postsynaptic region. ImageJ software was used to measure the length of the synapse contact and count synapses. Scale bar: 0.5 μm. (**D**) PSD-95 (red) and synaptophysin (green) antibodies were used to stain synapses in the cortical in 2-month-old mice. Graphs depict relative integrated density of PSD-95 and synaptophysin. Scale bar: 2 μm. (**E**) Analysis of SynGO cell components shows overlapping genes represented in a sector diagram. The central sector corresponds to the highest-level term, “synapse,” with subsequent outward sectors depicting its subclasses. (**F**) Representative images showing spine density in neurons transfected with WT or variant constructs. The graphs display statistical analyses of total, immature, mushroom, and stubby spine densities. Scale bar: 5 μm. *P* values were determined from 1-way ANOVA with Dunnett’s multiple comparisons test. (**A**, **C**, and **D**). *P* values were determined from a 2-tailed unpaired *t* test. *P < 0.05, **P < 0.01, ***P < 0.001, ****P < 0.0001. Each biological replicate (mouse) is color-coded; gray dots show individual data points, and colored dots indicate the mean per mouse. Data are reported as mean ± SEM.

The occurrence of NDD is closely related to changes in the number and morphology of dendritic spines. To investigate the effect of *Ints6* on the development of dendritic spines in neurons, we analyzed dendritic spines in cHET mice crossed with Thy1 EGFP mice with EGFP expression under the control of a modified Thy1 promoter region, which contains the sequences required for neuronal expression but lacks the sequences required for expression in non-neural cells (34). At 5 weeks old, cHET mice displayed a decrease in mushroom and stubby-type dendritic spines and an increase in filopodium and thin pseudopodia spines compared with WT mice (Figure 7A). Further analysis using Golgi staining showed a reduction in overall dendritic spines and mature mushroom spines in the cortex layer 2/3 neurons of cHET mice, yet the total dendritic spine count was unaffected (Figure 7B). Considering that the abnormal behaviors observed in *Ints6* cHET mice are associated with hippocampal function, we also examined dendritic spines in the hippocampus and found a similar phenotype to that observed in the cortex (Supplemental Figure 11, A and B).

To further confirm this phenotype, we performed in utero electroporation with GFP and pLKO.1 shRNA (NC) or *Ints6* shRNA plasmids in WT E14.5 mouse embryos. We extracted and cultured the primary neurons from E16.5 embryos and performed fluorescence staining at 18 days in vitro. Consistent with the findings of the in vivo analysis, *Ints6* knockdown substantially reduced the total number of dendritic spines and specifically decreased mushroom-shaped spines compared with the control group (Supplemental Figure 11C). To validate whether the deficits in spine maturation are related to the PP2A–RNAPII axis, we treated primary neurons from NC and *Ints6* knockdown mice with a CDK9i. The results showed that CDK9i treatment effectively reversed the abnormal spine phenotype induced by *Ints6* knockdown (Supplemental Figure 11D).

To further investigate the role of *Ints6* in synaptic development, we conducted electron microscopy and immunofluorescence analysis of cortical synapses in WT and cHET mice. Electron microscopy revealed a marked reduction in cortical synapse density in cHET mice. However, synapse contact length did not change compared with WT mice (Figure 7C). Similarly, immunofluorescence staining for PSD95, a postsynaptic marker, and synaptophysin, a presynaptic marker, in layer 2/3 of the cortex in 2-month-old mice had decreased PSD95 and synaptophysin puncta, along with reduced colocalization of these markers, indicating structural synaptic deficits (Figure 7D). Because synaptic reductions may be attributed to changes in neuronal numbers, we performed NeuN staining of the cortical layer 2/3 of 2-month-old mice. The results revealed no significant difference in neuronal numbers, suggesting the synaptic deficits are not due to neuronal loss (Supplemental Figure 12).

To explore the molecular mechanisms underlying *Ints6*’s effect on synaptic development, RNA-Seq was performed on cortical tissue from 2-month-old *Ints6* cHET mice. Synaptic Gene Ontology and Annotations (SynGO) analysis of the DEGs revealed significant enrichment in the synapse, postsynapse, postsynaptic cytoskeleton, and postsynaptic intermediate filament cytoskeleton (Figure 7E and Supplemental Table 8). Consistent with these findings, KEGG and GO enrichment analyses also highlighted significant involvement of synapse-related pathways (Supplemental Figure 13, A and B). To further strengthen the link between *Ints6* haploinsufficiency and synaptic dysfunction, we isolated synaptic fractions from WT and *Ints6* cHET mice and conducted proteomic analysis. The SynGO analysis of differentially expressed proteins again demonstrated significant enrichment in synapse-related pathways (Supplemental Figure 13C and Supplemental Table 9), emphasizing the disruption of key synaptic processes in *Ints6* cHET mice. These results indicate that *Inst6* is crucial for dendritic spine maturation.

To investigate whether NDD-related missense variants contribute to impaired dendritic spine maturation observed in the *Ints6* cHET mice, we introduced WT and NDD-related variant constructs into embryos via electroporation. Overexpression of *INTS6* WT plasmid markedly rescued the reduction of mushroom-shaped and stubby spines. However, none of the disorder-related de novo missense variants were able to restore spine maturation, indicating a loss-of-function effect of these missense variants in synapse development, which is consistent with the *INTS6* LGD variants identified in NDD (Figure 7F).

## Discussion

In this study, we combined approaches in clinical genetics, structural biology, mouse models, multi-omics, and functional assays to provide compelling evidence linking *INTS6* dysfunction to NDDs. Our findings further establish INTS6 as an essential component of the INTS complex, mediating neural progenitor proliferation, differentiation, cortical layer formation, and synaptic maturation, which contribute to the underlying pathogenesis of INTS-related NDD.

Our analysis of genomic data from large NDD cohorts and international multiple center collaborations identified 13 LGD variants from 15 families in *INTS6*, along with 8 de novo missense variants concentrated in or close to the VWFA domain. The pathogenicity of these missense variants is supported by structural modeling, which suggests that the variants disrupt critical protein-protein interactions or destabilize the structural integrity of INTS6, impairing its function within the INTS-PP2A complex. However, because these functional predictions have not been directly validated by experimental assays, we cannot conclusively confirm the predicted effects, which is a limitation of this study. Nevertheless, the observed inability of the missense variants to reduce RNAPII Ser2 phosphorylation in immunoblotting assays and to rescue dendritic spine maturation in electroporation assays aligns with the predicted structural disruptions, further supporting their pathogenicity and loss-of-function effects. Phenotypically, individuals with *INTS6* variants exhibit overlapping features, including language and motor delays, ASD, intellectual disabilities, sleep disturbances, and ADHD. These findings underscore the shared phenotypic spectrum of INTS6-associated NDDs and highlight its broad impact on neurodevelopment.

Using a cKO mouse model, we demonstrated that *Ints6* deletion disrupts cortical development, particularly affecting neurogenesis and cortical lamination. KO mice had a thickened VZ/SVZ and thinning of CP, with reduced differentiation and increased apoptosis of CP L6. These defects were associated with impaired RNAPII function and dysregulation of key cell cycle pathways. Furthermore, behavioral assays in cHET mice revealed impairments in social novelty preference, spatial memory, and hyperactivity, alongside structural and functional deficits in dendritic spines and synapses. Molecular studies linked these phenotypes to disruptions in RNAPII activity, PP2A regulation, and altered expression of synaptic genes.

INTS6 was originally identified as a tumor-suppressor protein (35–37), and it is now clear that several additional INTS subunits are misregulated or mutated in human cancers. The INTS complex is a multi-subunit protein assembly associated with RNAPII, which plays a crucial role in regulating transcriptional termination, RNA processing, and maintaining genomic stability. It exerts its regulatory function through 2 main activities: RNA endonuclease activity mediated by INTS11 and protein phosphatase activity facilitated by INTS6 and PP2A. INTS11 cleaves nascent RNA at paused RNAPII sites, preventing nonproductive elongation and ensuring precise transcriptional regulation. Meanwhile, INTS6 recruits PP2A to transcription sites, counteracting the activity of cyclin-dependent kinase CDK9, which controls release of an RNAPII pause. This dephosphorylation mechanism ensures that transcription progresses only under appropriate conditions, preserving a balance between gene activation and repression. Disruptions in this balance, such as through mutations in *INTS6* or *INTS11*, lead to aberrant transcriptional elongation, resistance to CDK9 inhibition, and amplified oncogenic transcriptional responses, linking the INTS complex to both transcriptional regulation and tumor susceptibility (33, 38). In our study, the CDK9 inhibitor effectively reversed the neural development and synaptic maturation defects caused by Ints6 deficiency. However, this rescue experiment has certain limitations. Notably, we did not perform behavioral assessments in mice to evaluate whether modulation of RNAPII activity translates into functional improvements, which is a critical step in validating the therapeutic potential of this approach. Despite this, our findings suggest modulating the balance between kinase and phosphatase activities may represent a promising strategy for treating specific NDDs associated with disrupted INTS function.

The cryogenic electron microscopy structures of the INTS complex have provided substantial insights into its mechanism, revealing 3 distinct states: pretermination, post-termination, and inactive (39). In the pretermination state, the scorpion-like tail module of the INTS complex positions INTS11 for RNA cleavage while disrupting the DRB sensitivity-inducing factor clamp and upstream DNA, triggering the collapse of the transcription bubble and the release of DNA from RNAPII. INTS3 prevents RNAPII from rebinding, ensuring proper termination and preparing the complex for the next cycle. The involvement of INTS6 in regulating the inactive state by occupying the PP2A catalytic site ensures the complex remains dormant until reactivation is needed. This mechanism of transcription termination is distinct from the torpedo model of 5′-3′ exonuclease activity (e.g., XRN2), because it operates independently of XRN2 in many noncoding loci, with INTS11 acting as a dual-function nuclease to degrade RNA and push RNAPII forward (40, 41).

Our RIP-Seq and ChIP-Seq data revealed increased binding of RNAPII to both RNA and DNA, suggesting altered transcriptional dynamics. RNA-Seq further identified upregulated and downregulated genes, indicating a complex transcriptional response associated with INTS6 deficiency. These findings align with previous studies in human cells, in which depletion of INTS subunits led to both up- and downregulation of protein-coding genes, as observed in HeLa cells depleted of INTS11 (*n* = 667 upregulated and 616 downregulated genes) (42, 43). This dual effect suggests that INTS may play a complex and context-dependent role in transcriptional regulation, which contrasts with simpler models observed in *Drosophila* and *Caenorhabditis* *elegan*s (44). Our data are consistent with the hypothesis that INTS is crucial for robust transcriptional induction of certain human protein-coding genes, particularly immediate-early genes. Previous studies have shown that INTS1 and INTS11 are recruited to enhancers of these genes, with EGF stimulation enhancing INTS binding in an ERK1/2-dependent manner (45, 46). When INTS1 or INTS11 is depleted, transcriptional induction of these genes is significantly diminished, which mirrors the transcriptional dysregulation observed in our study. The increased RNAPII binding and altered gene expression in our data suggest INTS6 may similarly influence transcriptional initiation and elongation at specific loci, potentially through mechanisms involving both RNAPII pausing and enhancer regulation. Our results show that the deletion of INTS6 not only affects the proliferation of neural stem cells but also leads to substantial apoptosis of CP L6 cells in *Inst6* cKO mice. This observation suggests INTS6 may regulate the expression of different genes at various developmental stages and in different cell types, contributing to distinct cellular outcomes. These results emphasize the need for further investigation into how INTS6 and other INTS subunits interact with transcriptional machinery to regulate gene expression, especially under stress or developmental conditions.

The INTS complex plays an indispensable role in early development, tissue morphogenesis, and cell differentiation. In early embryonic stages, it is essential for the regulation of RNAPII pausing and termination. For example, *Ints1* deletion in mouse embryos results in early lethality, likely due to the destabilization of the entire complex (47). Similarly, mutations in *Drosophila* INTS core components result in mid- to late-larval lethality (48). In zebrafish, mutations in *Ints6* disrupt gastrulation, highlighting the essential role of the INTS in developmental stages through the regulation of dorsal organizer genes and the maintenance of proper transcriptional pausing (49). INTS also plays a role in stem cell maintenance and tissue regeneration, with studies in planarian flatworms demonstrating the importance of INTS components for stem cell function and tissue renewal. In mice, INTS is involved in adipocyte differentiation, underscoring its broad biological relevance (50). In neural development, the INTS complex is particularly crucial in regulating neuronal migration, differentiation, and progenitor cell maintenance. In mouse neuronal progenitor cells, INTS coordinates cortical neuron migration through interactions with ZFP609 and NIPBL, which regulate transcriptional processes critical for neuron positioning and development (51). In *Drosophila*, INTS prevents dedifferentiation of intermediate neural progenitors, thereby ensuring proper commitment to terminal cell fates (52).

In humans, mutations in INTS subunits, including *INTS1* (52, 53), *INTS8* (54), *INTS11* (55) and *INTS13* (56), have been linked to severe NDDs characterized by developmental delays, intellectual disabilities, and structural brain abnormalities. INTS1 and INTS11 are part of the catalytic modules and are essential for RNA cleavage and transcription termination. Mutations in these subunits cause severe cognitive and motor impairments, including cognitive delay, absence of speech, cataracts, glaucoma, and facial dysmorphism (INTS1) or growth restriction, microcephaly, and cerebellar atrophy (INTS11). These defects arise due to improper RNA processing and transcriptional dysregulation. In contrast, INTS8 and INTS6 are involved in the phosphatase module and regulate the recruitment of PP2A to transcription sites, ensuring proper dephosphorylation of RNAPII and transcription factors. Variants in *INTS8* result in severe cognitive delay, speech absence, and motor impairment, whereas monoallelic variants in *INTS6* lead to milder phenotypes, including speech–language problems, motor delays, and intellectual disability. This suggests that INTS6 may retain partial function despite the monoallelic mutation. Finally, INTS13, part of the enhancer module, does not affect RNA cleavage or phosphatase activity but regulates enhancer-driven gene expression. Variants in *INTS13* result in oral, facial, and digital anomalies and speech abnormalities, highlighting its role in the spatial regulation of transcription. The phenotypes associated with *INTS6* mutations highlight the critical role of the Int–PP2A module and Pol II pausing in human development. Notably, point mutations in PP2A-A have been linked to a broad spectrum of NDDs (56–58). We hypothesize that transcriptional dysregulation in developing embryos with PP2A-A mutations could be a major pathogenic factor in these disorders. Future studies should focus on understanding the specific contributions of disrupting canonical PP2A ternary complexes versus INTS -bound PP2A to better elucidate the molecular mechanisms underlying these developmental defects.

Unlike dominant mutations in *INTS6*, biallelic mutations in other *INTS* genes, such as *INTS11*, are associated with neurodevelopmental or neurological disorders. This difference may arise because genes like *INTS11*, which serve as the catalytic core of the INTS complex, retain sufficient activity at reduced dosages or are buffered by compensatory mechanisms. In contrast, INTS6 likely plays a unique structural or regulatory role, making its function highly dosage sensitive. Supporting this, neuronal knockdown of *Ints11* produces no apparent phenotype (9), whereas *Ints6* knockdown results in substantial neural abnormalities.

In addition to its well-established role in the phosphatase module of the INTS complex, INTS6 may also contribute to NDDs through other mechanisms. Recent studies have highlighted direct interactions between INTS6 and INTS3, suggesting INTS6 may function in ways that are independent of its role in the INTS complex. Specifically, immunoprecipitation experiments and crystal structure analyses have shown that the C-terminal domain of INTS3 mediates interaction with INTS6, possibly facilitating the formation of a heterotrimeric complex composed of INTS3/INTS6/hSSB1 (59). This interaction may involve multimerization, pointing to a potential alternative function of INTS6 outside the classical transcriptional regulation pathway. This suggests INTS6 could have roles in cellular processes beyond transcriptional termination, such as genomic stability and DNA repair, through its interactions with hSSB1, a factor involved in DNA damage response (60, 61). Furthermore, INTS6 has been shown to regulate dorsoventral patterning during development by modulating key signaling pathways. For instance, INTS6 disrupts the expression of critical signaling factors, including BMP ligands and mediators of the Wnt signaling pathway, which are essential for proper patterning during embryogenesis (35, 49). These findings underscore the potential role of INTS6 in developmental processes that go beyond its canonical function in transcriptional regulation.

## Methods

### Sex as a biological variant.

Our study exclusively examined male mice because the *INTS6* variants are associated with a potential male predominance.

### De novo variants in INTS1–15 and burden analysis.

De novo likely gene-disruptive and missense variants in the coding regions of INTS1–15 were identified from 32,323 individuals with ASD and 31,085 individuals with intellectual and developmental disabilities across 5 whole-exome and genome sequencing studies. These included a large unpublished ASD cohort, the SPARK cohort (12), which comprises 20,646 ASD individuals from 18,405 families with whole exome sequencing/whole genome sequencing data passing quality control; and 4 published cohorts: the SSC cohort (13), the Autism Sequencing Consortium cohort (14), the MSSNG cohort (15), and the Deciphering Developmental Disorders cohort (2). Potential duplicate samples were excluded if they had the same identifier or carried identical variant(s) originating from related cohorts (e.g., SPARK, SSC). The variants were re-annotated using the ANNOVAR tool. To evaluate the burden of de novo coding variants in INTS1–15, we performed an analysis using *DenovolyzeR* (16), a probabilistic model with default settings. This approach calculates the expected number of de novo variants in a given population based on the mutability of a gene and the number of sequenced probands, comparing it with the observed number using a Poisson framework.

### Animal.

*Ints6*^flox/flox^ mice were generated by Cyagen Biotechnology using the CRISPR-Cas9 method, following the strategy outlined in Supplemental Figure 2A. The strategies for selecting the KO regions were as follows: ensure than (a) the sequences of the homology arm and the cKO region were aligned to each other without tandem repeats for PCR screening or sequencing analysis; (b) the homology arm sequence and cKO region had suitable GC content for PCR screening or sequencing analysis; and (c) the 3,000 bp upstream of the cKO region and the 3,000 bp downstream of the cKO region did not show marked similarity to the genome. Considering these considerations, E5 and E6 were suitable KO fragments. Nestin-Cre mice and Thy1-GFP transgenic mice were provided by Yuan Ling (Central South University), and C57BL/6J mice were obtained from the Medical Genetics Experimental Animal Center of Central South University. Age- and sex-matched littermate pairs were used in the experiments to ensure consistency. Animals were housed in acrylic cages with ad libitum access to water and food and maintained on a 12-hour light/dark cycle. Environmental conditions were controlled at 22 ± 2°C and 45%–55% humidity.

For complete methods, see Supplemental Data Methods.

### Statistics.

All experiments were performed at least 3 times. Appropriate statistical tests were applied. Data are presented as mean ± SEM. For 2-group comparisons, *P* values were determined from a 2-tailed unpaired (or paired) *t* test for normally distributed data and a Mann-Whitney test for non-normally distributed data. For comparisons among more than 2 groups, 1-way ANOVA with Dunnett’s multiple comparisons test was used for normally distributed data, and the Kruskal-Wallis test with Dunnett’s multiple comparisons test was used for non-normally distributed data. For paired data, the Friedman test with Dunn’s multiple comparisons test was applied for non-normally distributed data. Two-way ANOVA with Dunnett’s or Bonferroni’s multiple comparisons test was used for the analysis of interaction effects between 2 factors. *P* < 0.05 was considered statistically significant.

### Study approval.

Written informed consent was obtained from study participants or their parents or legal guardians, in line with local IRB requirements at the time of collection. The IRB of the Central South University approved this study (approval no. IRB 2022-1-3). All animal procedures were approved by the Animal Ethics Committee of Central South University and conducted in accordance with institutional guidelines to minimize animal suffering and adhere to the 3R (Replacement, Reduction and Refinement) principle. All animal experiments complied with all relevant ethical regulations and were approved by the IRB of Central South University (approval no. IRB 2022-2-3).

### Data availability.

All raw sequencing data generated in this study have been deposited in the National Center for Biotechnology Information Sequence Read Archive. The RNA-Seq data sets are available under BioProject accession number PRJNA1308459, and the CUT&Tag-Seq and RIP-Seq data sets are available under BioProject accession number PRJNA1294551. Approved researchers can obtain the SSC population data set described in this study (https://www.sfari.org/resource/simons-simplex- collection/) and the SPARK population data set described in this study (https:// www.sfari.org/resource/spark/) by applying at https://base.sfari.org The data values of all graphs and values behind any reports means in the manuscript are provided in the Supporting Data file.

## Author contributions

HG, JT, KX, XP, and XJ designed and conceived this study. XP, HW, XJ, JC, XD, CQ, MH, and HH performed the mouse behavioral and neurogenesis analyses. XJ, XZ, and ST performed genomic analysis and interpreted the genotype and phenotype data. WZ, YZ, Zhengmao Hu, QP, FL, G Chen, JL, and Zhangxue Hu helped with data interpretation. HG, JT, and KX supervised the work. XP, XJ, JT, HG, and KX wrote and revised the manuscript. The order of the 2 first authors’ names was determined by joint decision of these authors. Other authors, including IP, AK, FJK, AR, RC, CS, JP, BC, BI, SM, GN, AMG, FF, LM, LC, GB, GČ, BP, AS, KB, JAMA, CvRA, DB, KHK, TB, ZS, RAJ, and EAE, contributed and interpreted the genetic and clinical data recruited from an international collaborative network. All authors commented on the manuscript and approved the final manuscript.

## Funding support

- STI 2030-Major Project, 2021ZD0201704, to HG.
- National Natural Science Foundation of China, 82222025 and 32271141, to HG; 82130043, 82330035, and 82361138573, to KX; 82160219, to WZ; and 82401388, to XJ.
- National Key Research and Development Program of China, 2021YFA0805200, to ZH and JT.
- Hunan Provincial grants, 2023RC1020 and 2023SK2084 to HG; 2021SK1010 and 2023SK2114 to KX; and 2024JJ6545 to XJ.
- Jiangxi Province Key Research and Development Project, 20232BBG70023, to YZ.
- Xingdian Project of Yunnan Province, XDYC-QNRC-2022-0267, to WZ.
- The REDIA study from the French Ministry of Health, PHRC-I 18-38, to GN.
- Undergraduate Training Programs for Innovation and Entrepreneurship of CSU, X202410533687 to HH.

## Supplementary Material

Supplemental data

Unedited blot and gel images

Supplemental tables 1-9

Supporting data values

## Version 1 09/18/2025

In-Press Preview

## Version 2 11/17/2025

Electronic publication
