# Supplementary material for: Disrupting integrator complex subunit INTS6 causes neurodevelopmental disorders and impairs neurogenesis and synapse development
Source: J Clin Invest. 2025 Sep 18;135(22):e191729. doi: 10.1172/JCI191729 (PMC12618080; doi:10.1172/JCI191729)
Supplement: Supplemental data [file jci-135-191729-s236.pdf]

# Supplementary Data Figures

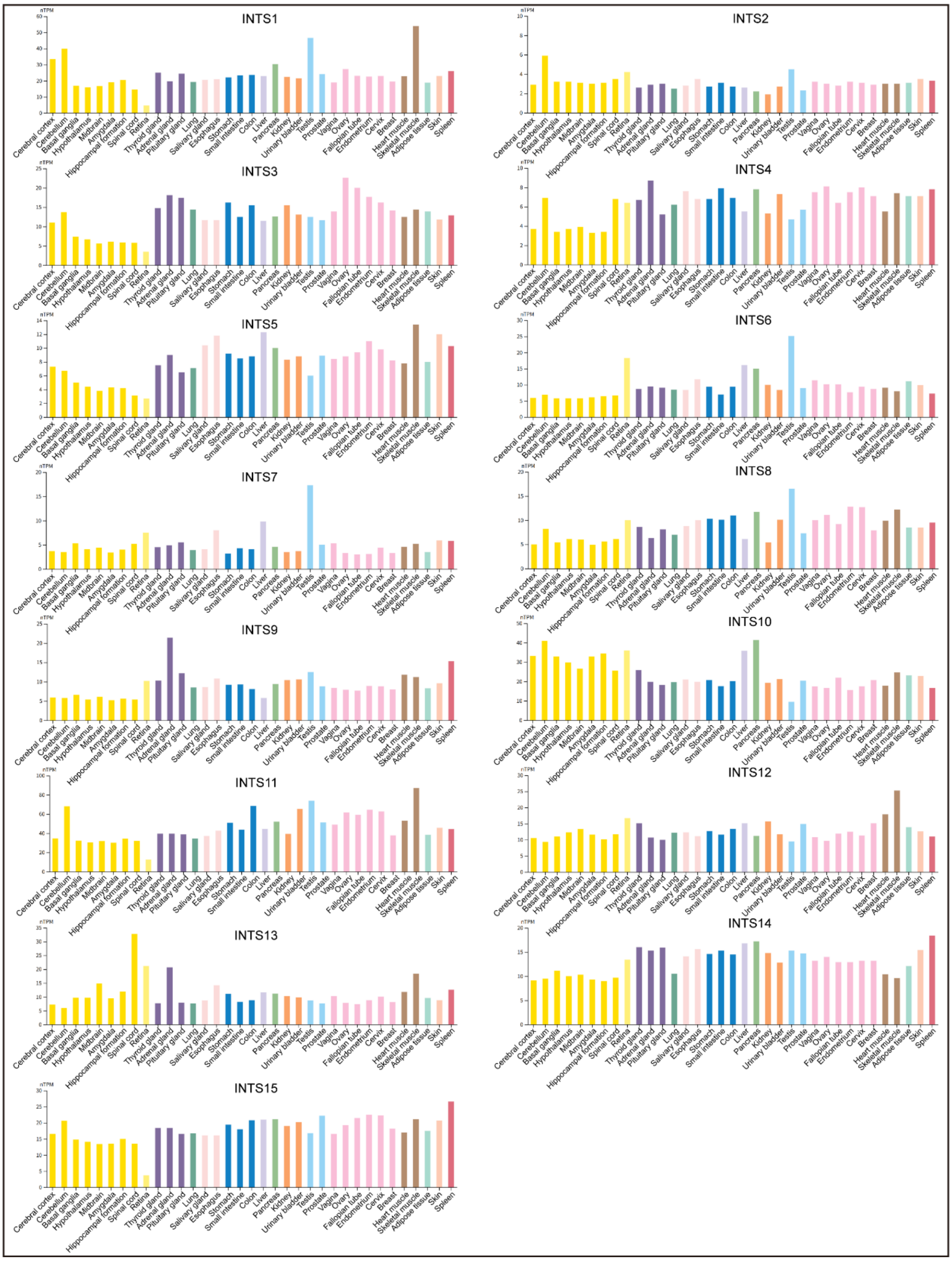


**Figure S1 Expression patterns of INTS1-15 in tissues.** RNA sequencing data from GTEx showing the expression patterns of INTS genes across various tissues.

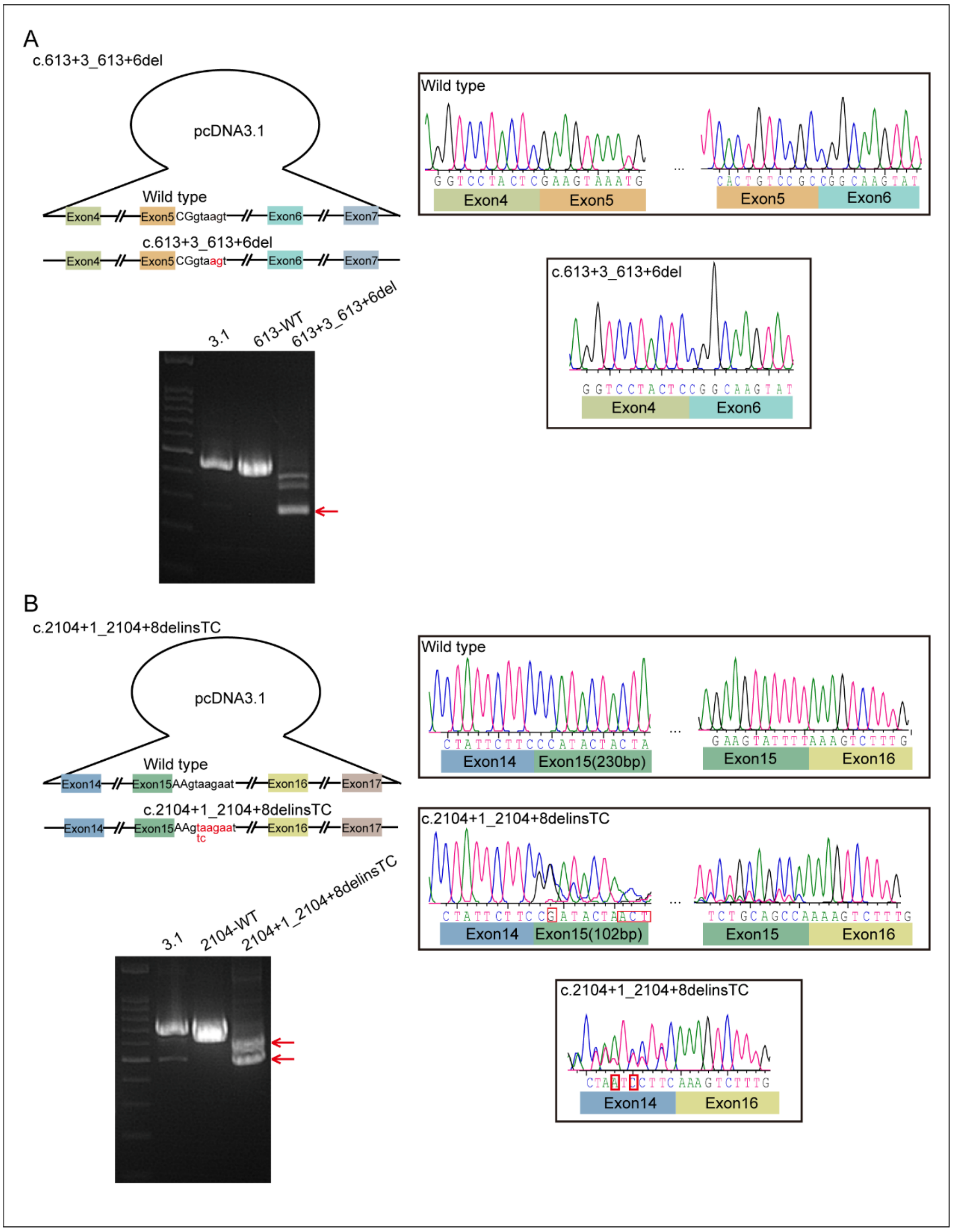


**Figure S2 Minigene constructs and *in vitro* splicing results for 2 de novo splice-altering variants.** (**A**) Schematic and analysis of the c.613+3_613+6del minigene in pcDNA3.1. This construct includes 321 bp downstream of intron 3, intact exons 4 through 7, and flanking intronic regions up to 327 bp. Below, agarose gel electrophoresis shows RT-PCR products for both wild-type and mutant transcripts, highlighting mutant-specific abnormal splicing indicated by the red arrow. Sequencing chromatograms on the right compare normal and aberrant splice variants. (**B**) Diagram and analysis of the c.2104+1_2104+8delinsTC minigene in pcDNA3.1. This vector encompasses 261 bp downstream of intron 13 through exon 17 with full intronic sequences and adjacent intronic regions up to 406 bp. Agarose gel electrophoresis of RT-PCR products illustrates the normal and mutant transcripts, with abnormal splicing in the mutant denoted by a red arrow. Sequencing results on the right detail the wild-type and altered splicing patterns.

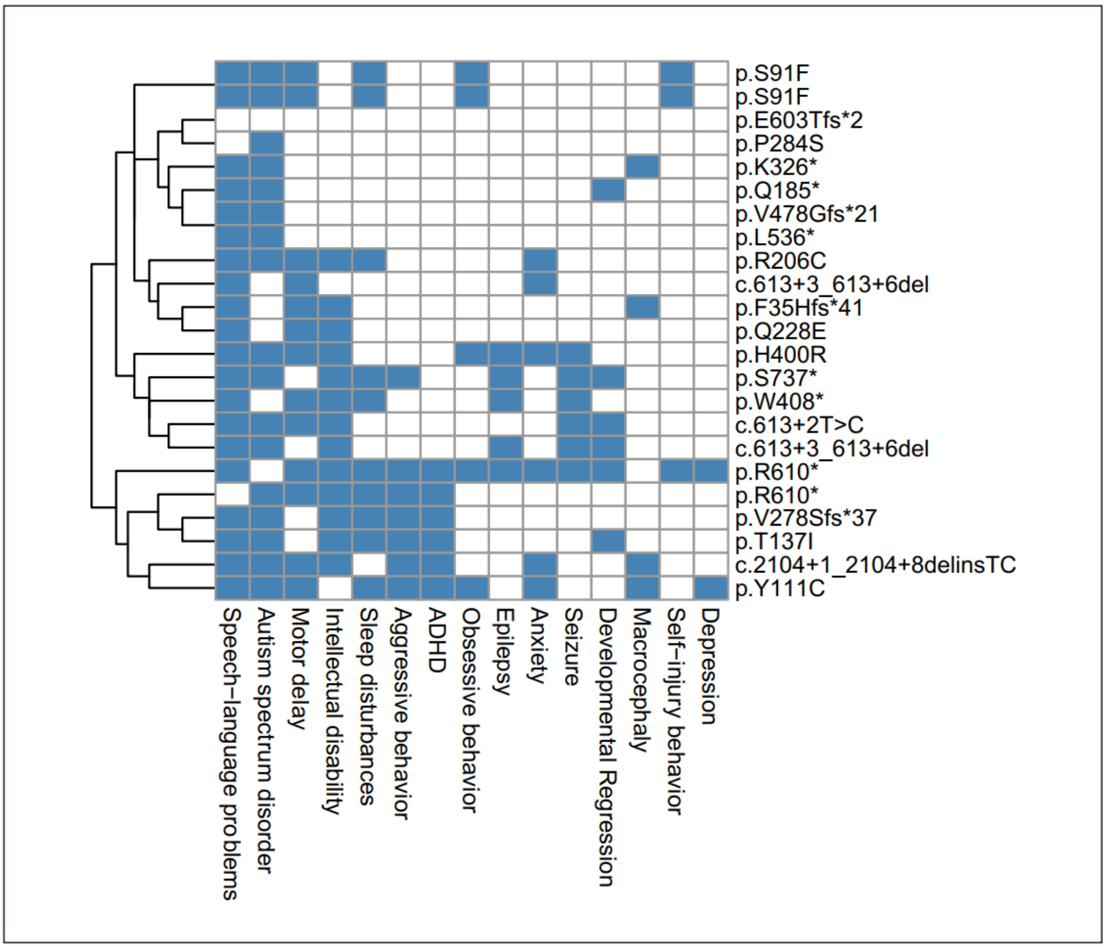


**Figure S3 Clustering analysis based on the clinical features.**

A

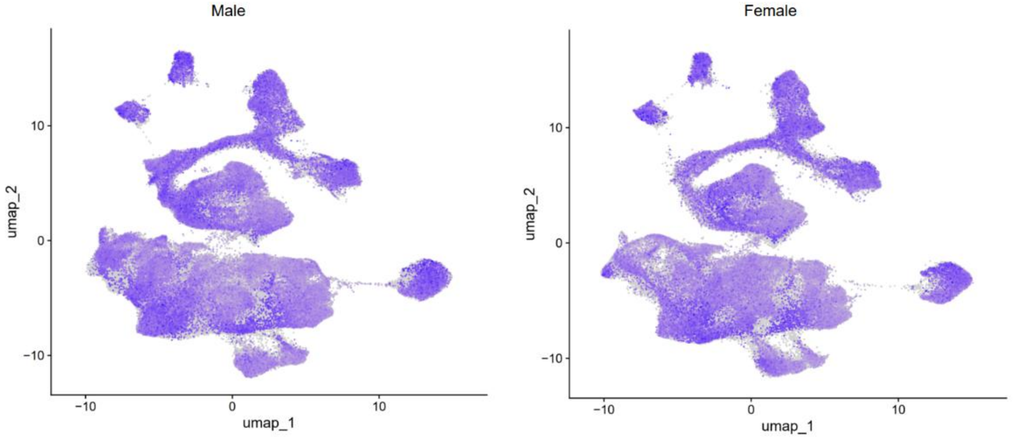


B

| cell type | OPC | ExNeu | OL | IN | MG | AST | VASC | GLIALPROG | Total |
|---|---|---|---|---|---|---|---|---|---|
| log2FC | -0.01856962 | 0.015626734 | 0.006775145 | 0.022489934 | -0.03079079 | 0.025131436 | -0.18496984 | -0.05050354 | -0.00435 |
| p_adjust | 1 | 5.09E-08 | 8.72E-14 | 1 | 1 | 0.000375561 | 1 | 1 | 1 |

**Figure S4 Expression patterns of INTS6 in male and female.** (**A**) UMAP plot of single-cell RNA sequencing data showing the expression patterns of INTS6 in male and female across distinct cell types. (**B**) Flod change and statistic analysis of INTS6 between male and female.

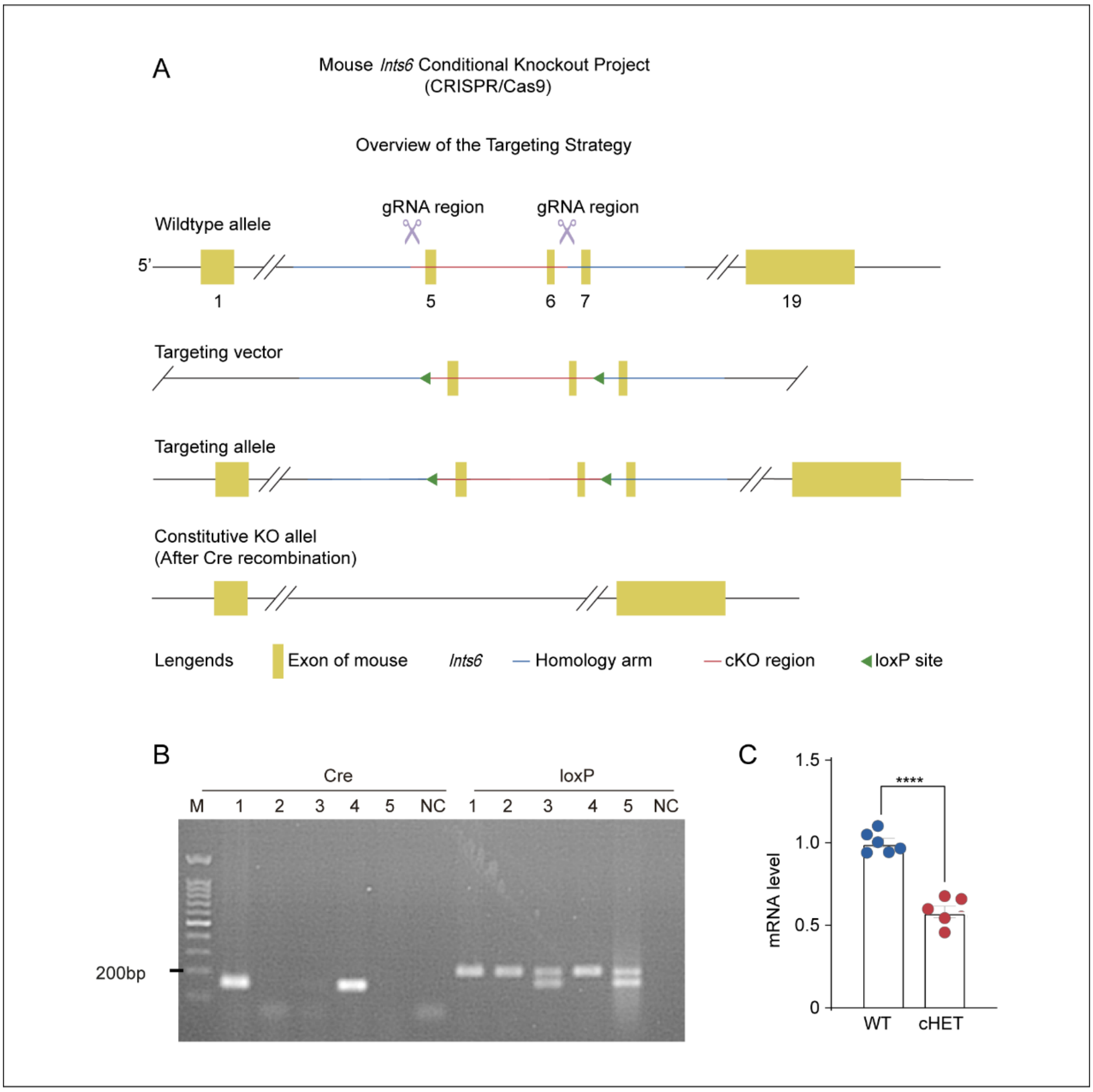


**Figure S5 Generation and Validation of Conditional *Ints6* Knockout in Mice Using CRISPR/Cas9 Technology.** (**A**) Schematic representation of the strategy for the conditional knockout of the mouse Ints6 gene using CRISPR/Cas9. The diagram illustrates the wildtype allele, the targeting vector with guide RNA (gRNA) sites, the targeted allele before and after Cre-lox recombination leading to the constitutive knockout (KO) allele. (**B**) Gel electrophoresis for genotyping of Ints6 in modified mice. The left image displays nestin-Cre identification with a 150 bp band, indicating the presence of Cre recombinase. The right image shows loxP site identification, with Ints6$^{fl/fl}$ depicted by a 204 bp band and Ints6$^{fl/wt}$ by both 154 bp and 204 bp bands. The mouse numbers 1, 2, 3, 4, and 5 correspond to genotypes cKO, WT, WT, cKO, and WT, respectively. (**C**): Quantitative analysis of Ints6 mRNA levels in brain tissue from wild-type and heterozygous (cHET) embryonic day 16.5 mice. Data are mean ± SEM. *P* values were determined from a 2-tailed unpaired *t* test. ***$P < 0.001$.

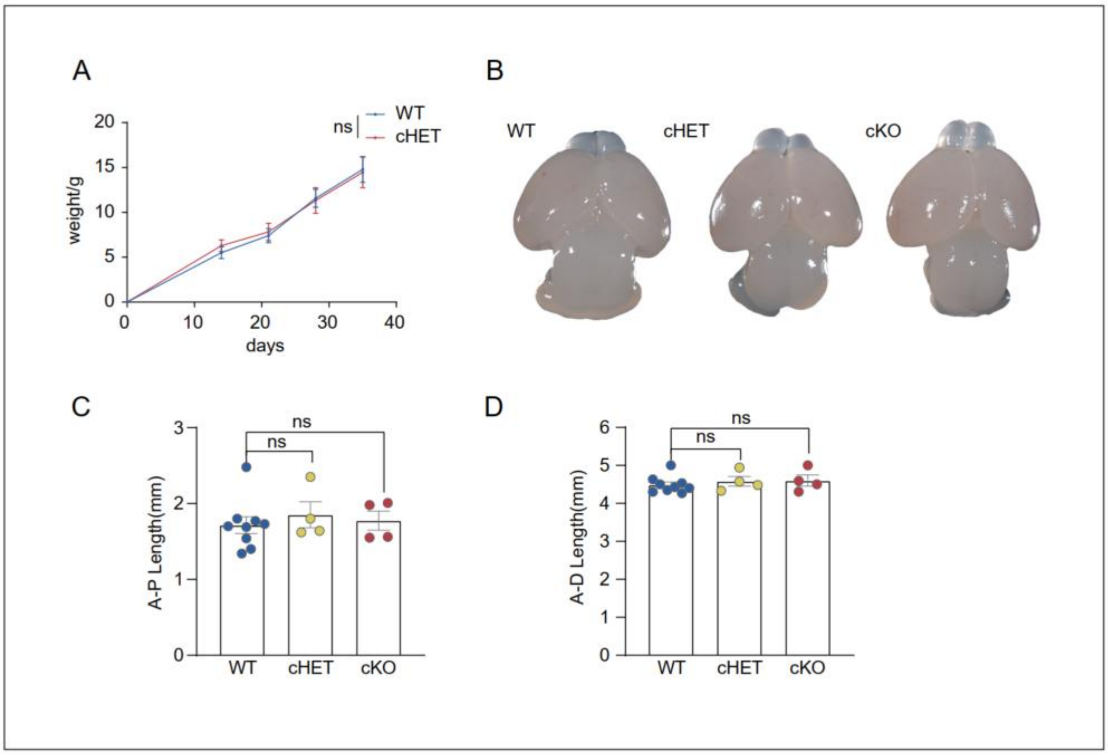


**Figure S6 Growth and Brain Development in *Ints6* Knockout Mice.** (**A**) Growth curves showing the weight progression over 35 days for wild-type (WT) and conditional heterozygous (cHET) *Ints6* knockout mice. Data are mean ± SEM. *P* values were determined from two-way ANOVA with Bonferroni's multiple comparisons test. (**B**) Representative images of E18.5 mouse brains from wild-type (WT), conditional heterozygous (cHET), and conditional knockout (cKO) mice, illustrating brain morphology. (**C**) Bar graph comparing the anterior-posterior (A-P) length of the cerebral cortex in WT, cHET, and cKO mice at E18.5. Data are mean ± SEM. *P* values were determined from Kruskal-Wallis with Dunnet's multiple comparisons test. (**D**) Bar graph comparing the anterior-dorsal (A-D) length of the cerebral cortex in WT, cHET, and cKO mice at E18.5. Data are mean ± SEM. *P* values were determined from one-way ANOVA with Dunnett's multiple comparisons test. ns = not significant.

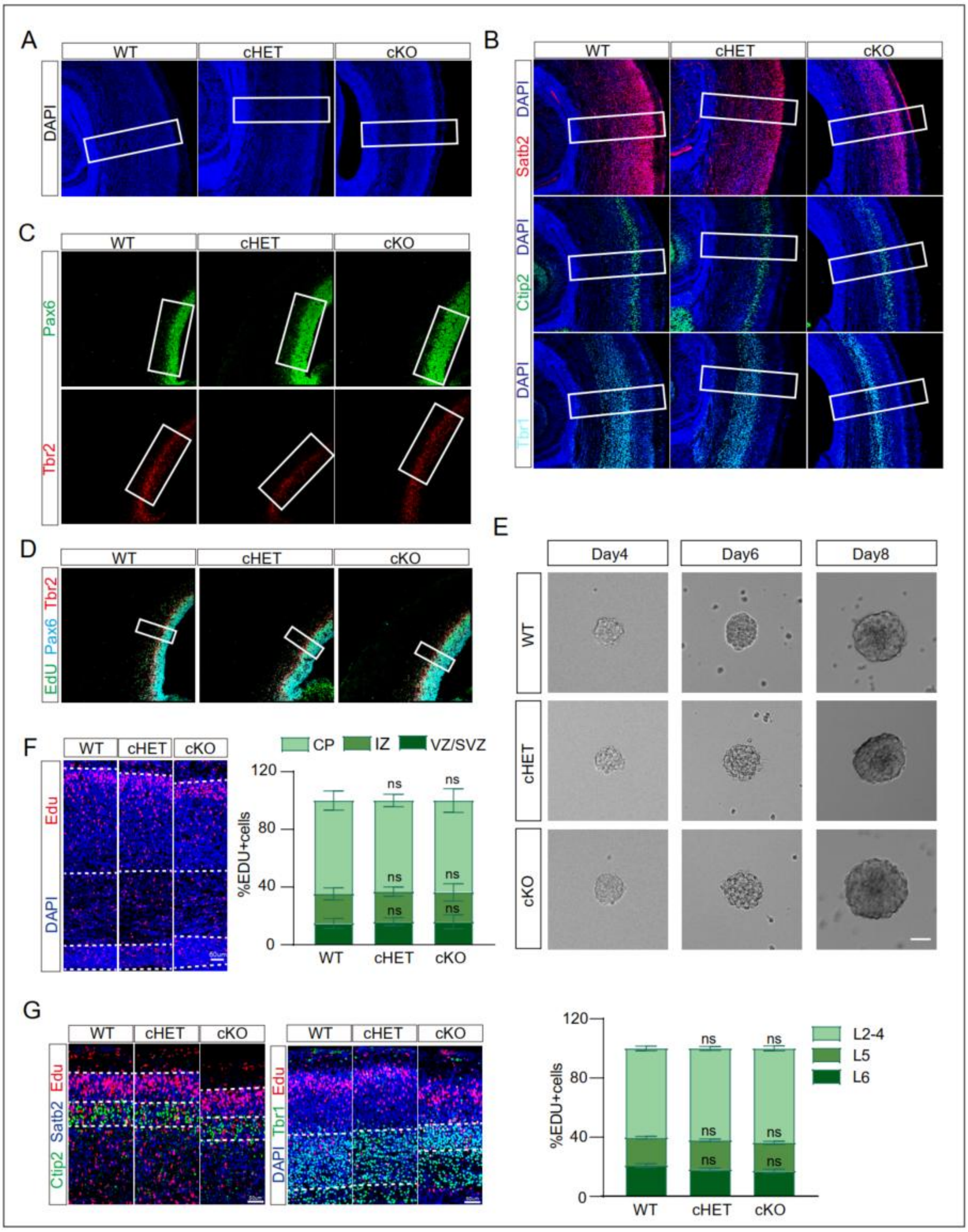


**Figure S7 Knockout of *Ints6* does not affect cortical neuron migration. (A)** The whole coronal slices corresponding to Figure 4A. **(B)** The whole coronal slices corresponding to Figure 4B. **(C)** The whole coronal slices corresponding to Figure 4D. **(D)** The whole coronal slices corresponding to Figure 4E. **(E)** The representative image corresponding to Figure 4F. (**F**) Immunofluorescence staining of EdU incorporation in the cortical regions of E14.5 to E18.5 WT (n=5), cHET (n=5) and cKO (n=5) INTS6 mice. Graph shows the percentage of EdU+ cells in the CP, IZ and VZ/SVZ. Scale bar: 50 μm. Data are mean ± SEM. *P* values were determined from Kruskal-Wallis with Dunnett's multiple comparisons test. (**G**) Immunofluorescence staining showing neuronal markers and EdU incorporation in E14.5 to E18.5 WT (n=5), cHET (n=5), and cKO (n=5) INTS6 mice cortex. Graphs display the percentage of EdU+ cells in layers 5 and 6. Scale bar: 50 μm. Data are mean ± SEM. *P* values were determined from one-way ANOVA with Dunnett's multiple comparisons test. ns= not significant; *$P$<0.05; **$P$<0.01; ***$P$<0.001; ****$P$<0.0001.

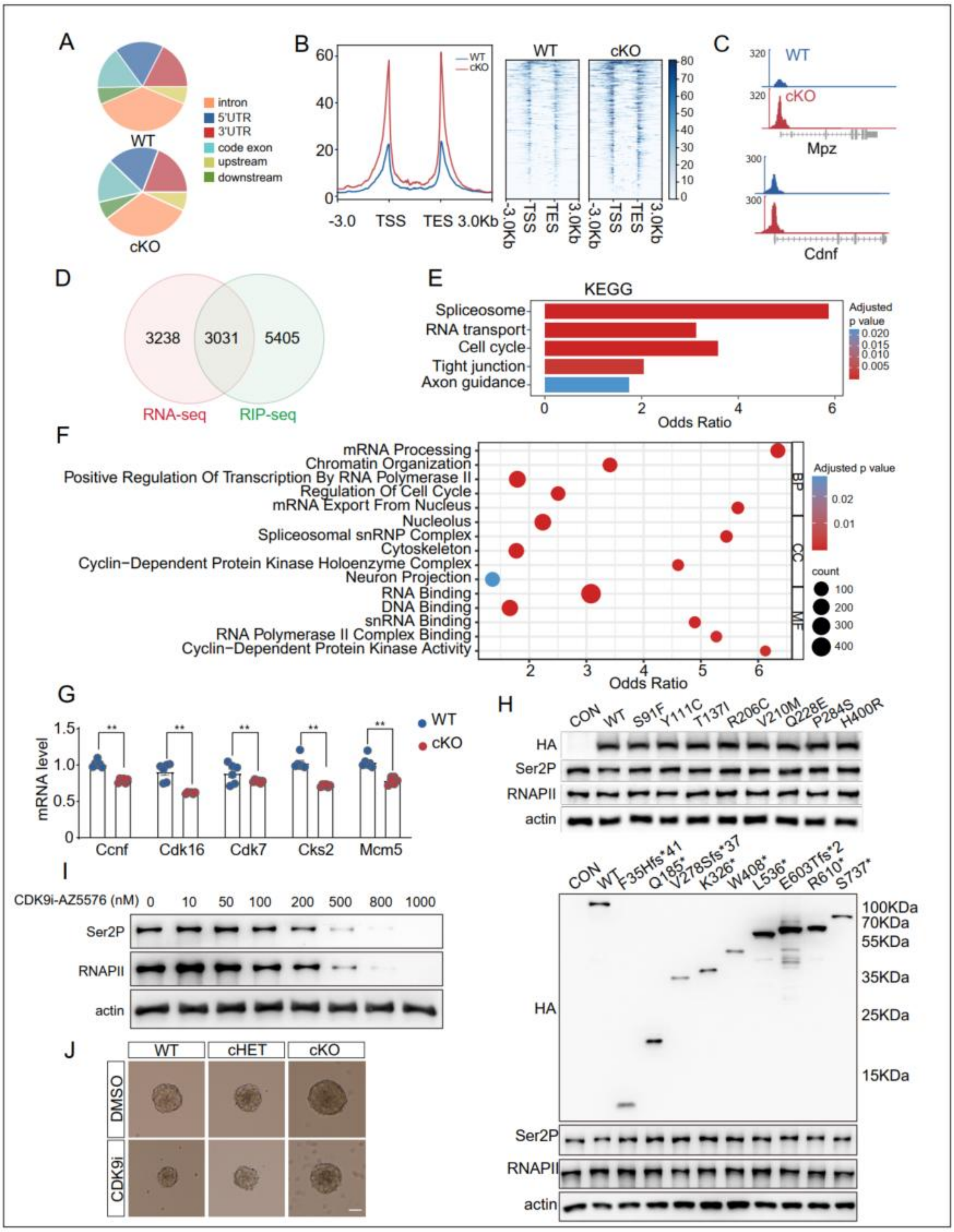


**Figure S8 *Ints6* deficiency disrupts PP2A-RNA polymerase II function.** (**A**) Pie chart distribution of RNA polymerase II across different genomic regions in RIP-seq from embryonic day 15.5 in WT and INTS6 cKO mice. (**B**) Spatial distribution and Heatmap of the distance of RNA polymerase II binding around the gene region of up-regulated genes of CUT&Tag and RNA-seq overlap genes in WT and cKO mice. The gradient blue-to-white color indicates high-to-low counts in the corresponding region. (**C**) Browser tracks of CUT&Tag profiles for up-regulated genes of embryonic development, comparing expression levels in WT and INTS6 cKO mice. (**D**) Venn diagram illustrating overlap between differential genes identified in RNAseq ($P$<0.05) and RIPseq ($P$<0.01) datasets. (**E and F**) KEGG and GO analysis of overlap gene from (**D**) data. (**G**) Real-time

PCR validation of the key genes related to cell cycle revealed by RIP-seq. Data are mean ± SEM. *P* values were determined from a 2-tailed unpaired Mann-Whitney test. (**H**) Western blot of total RNA polymerase II and Ser2-phosphorylated in HEK293T cells transfected with either WT, missense variants or LGD variants. (**I**) The inhibition of Ser2-phosphorylated by CDK9i in neuron. (**J**) Images of neurosphere treated by CDK9i of WT, cHET and cKO mice. $^{**}P<0.01$.

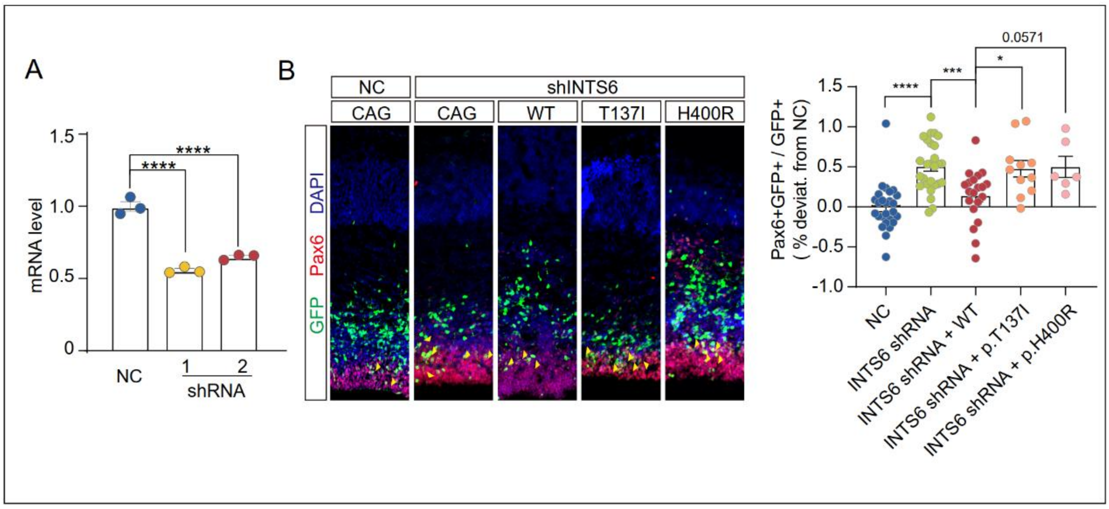


**Figure S9** ***INTS6*** **disorder-related variants impairs proliferation of neural progenitors. (A)** Quantitative PCR (qPCR) was performed to assess the knockdown efficiency of *Ints6* shRNA (shRNA1 and shRNA2) in N2a cells. Data are mean ± SEM. *P* values were determined from one-way ANOVA with Dunnett's multiple comparisons test. (**B**) Left: Images of E15.5 lateral ventricles of fetal mice electroporated at E13.5 with no targeting shRNA (NC); Ints6 shRNA; combination of Ints6 shRNA with WT; combination of Ints6 shRNA with p.T137I and p.H400R. Right: The percentage of Pax6+GFP+/GFP+ cells. Data are mean ± SEM. *P* values were determined from Kruskal-Wallis with Dunnett's multiple comparisons test. **P*<0.05; ****P*<0.001; *****P*<0.0001.

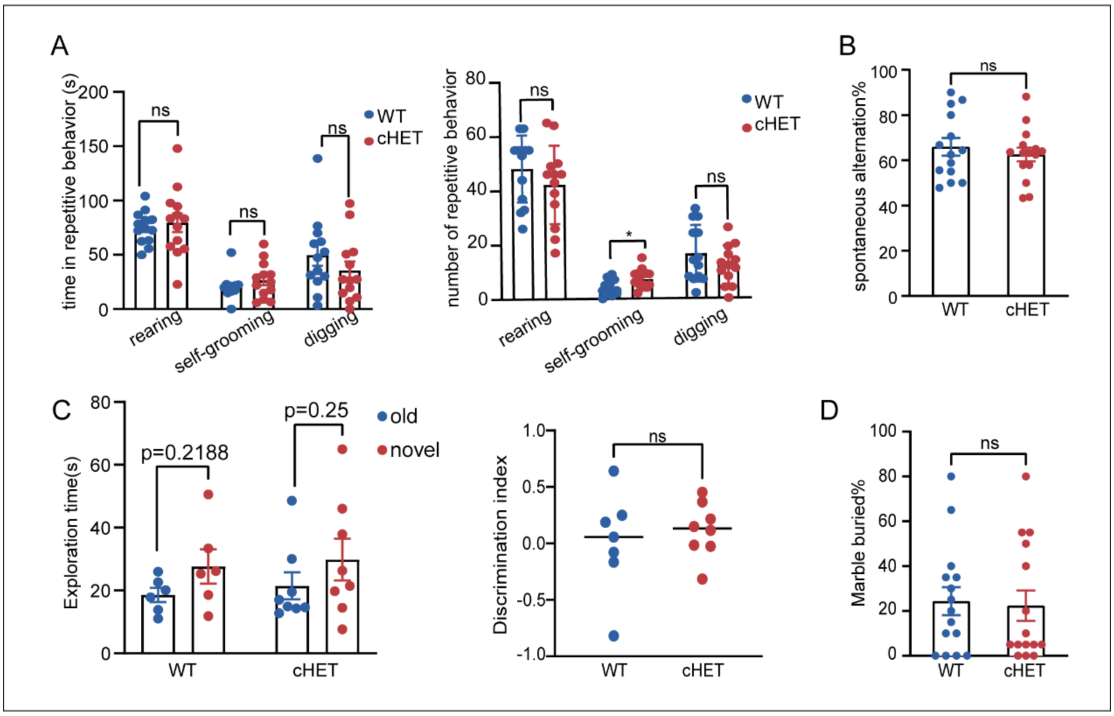


**Figure S10 Behavioral Assessments of Wild-Type and cHET Mice in Various Neurological Tests** (**A**) Quantitative analysis of restricted and repetitive behaviors observed over six minutes, including rearing, self-grooming, and digging. The right panel shows the counts of each behavior within the same timeframe, with a significant difference observed only in digging behavior. Data are mean ± SEM. *P* values were determined from a 2-tailed unpaired Mann-Whitney test. (**B**) Spontaneous alternation performance in a Y-maze shown as a percentage for both WT and cHET mice, indicating no significant difference in exploratory behavior. Data are mean ± SEM. *P* values were determined from a 2-tailed unpaired *t* test. (**C**) Object recognition test data displaying the exploration times for novel and familiar objects by WT and cHET mice, with no significant differences in recognition or novelty preference. Data are mean ± SEM. *P* values were determined from a 2-tailed paired and unpaired *t* test respectively. (**D**) Marble burying test comparing the percentage of marbles buried by wild-type (WT) and heterozygous (cHET) mice within 30 minutes, showing no significant differences. Data are mean ± SEM. *P* values were determined from a 2-tailed unpaired Mann-Whitney test. ns: not significant; **P*<0.05; ***P*<0.01; ****P*<0.001; *****P*<0.0001.

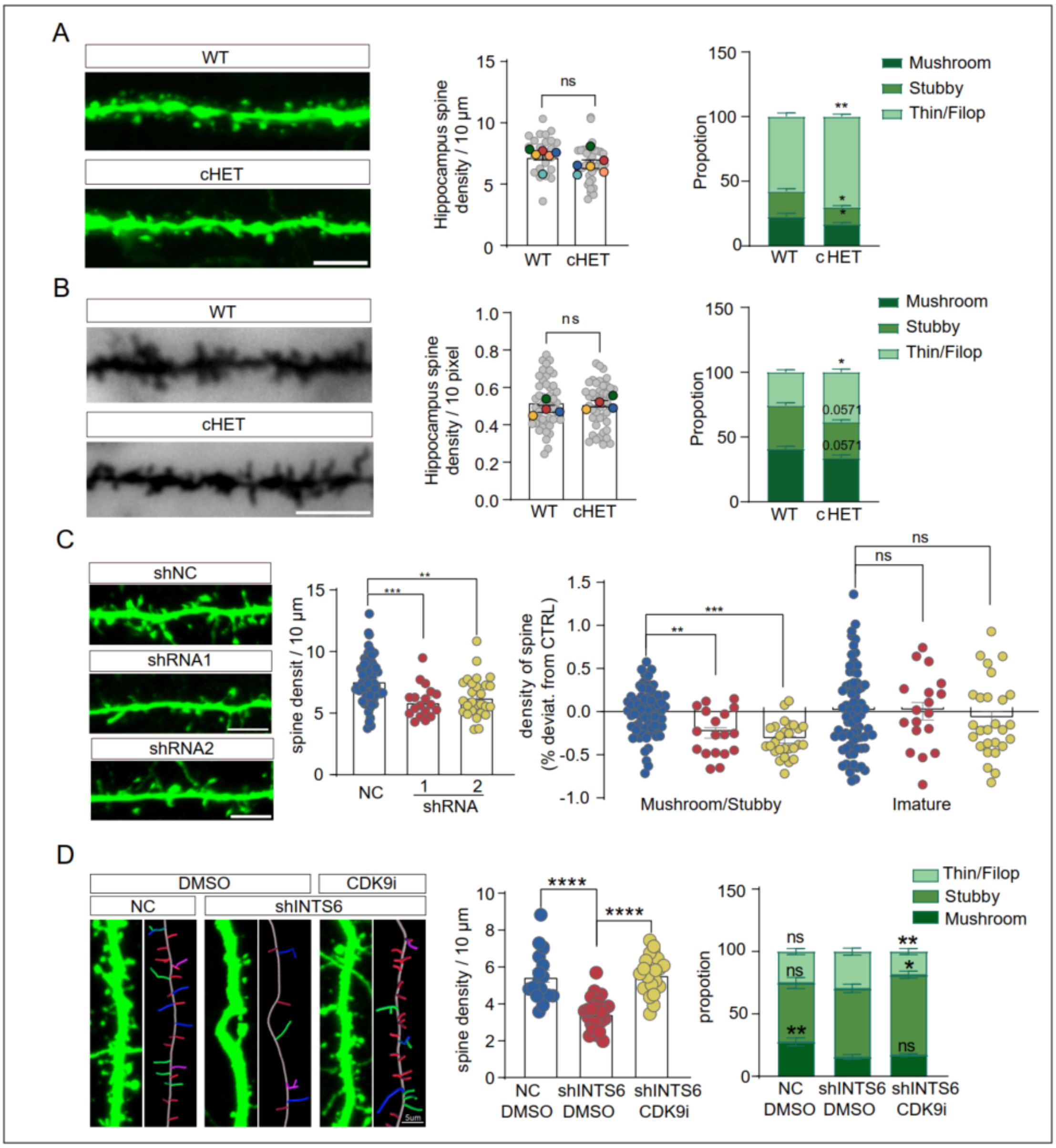


**Figure S11** ***Inst6*** **Knockdown Impairs Dendritic Spine Density and Maturation in Cultured Neurons Following in Utero Electroporation.** (**A**) Hippocampal dendritic spines from 5-week-old Thy1-GFP mice were stained with GFP antibody to visualize morphology and quantified by confocal microscopy, spine density and the three types (stubby, mushroom, and thin) were normalized to 10 μm dendritic length. Scale bar: 5 μm. Data are mean ± SEM. *P* values were determined from a 2-tailed unpaired Mann-Whitney and *t* test. (**B**) Golgi staining of dendritic spines in the hippocampus neurons of WT and cHET mice. Bar graph comparing percentages of spine types (filopodia, stubby, thin, mushroom) between WT and cHET, Scale bar: 100 pixel. Data are mean ± SEM. *P* values were determined from a 2-tailed unpaired Mann-Whitney and *t* test. (**C**) Representative dendritic spine images of primary neurons following in utero electroporation at E14.5 with pLKO.1 (NC) or INTS6 shRNA plasmids (shRNA1 and shRNA2), co-transfected with an EGFP plasmid. Immunofluorescence staining was performed at DIV18 and dendritic spine density and subtype analysis (mushroom/stubby and immature spines) were quantified using ImageJ. Scale bar = 5 μm. Data are mean ± SEM. *P* values were determined from one-way ANOVA with Dunnett's multiple comparisons test. (**D**) The density and maturity of dendritic spine cultured in

vitro treated by CDK9i. Data are mean ± SEM. *P* values were determined from one-way ANOVA and Kruskal-Wallis with Dunnett's multiple comparisons test. ns = not significant; $*P < 0.05$, $**P < 0.01$, $***P < 0.001$. Each biological replicate (mouse) is color-coded; gray dots show individual data point, and colored dots indicate the mean per mouse.

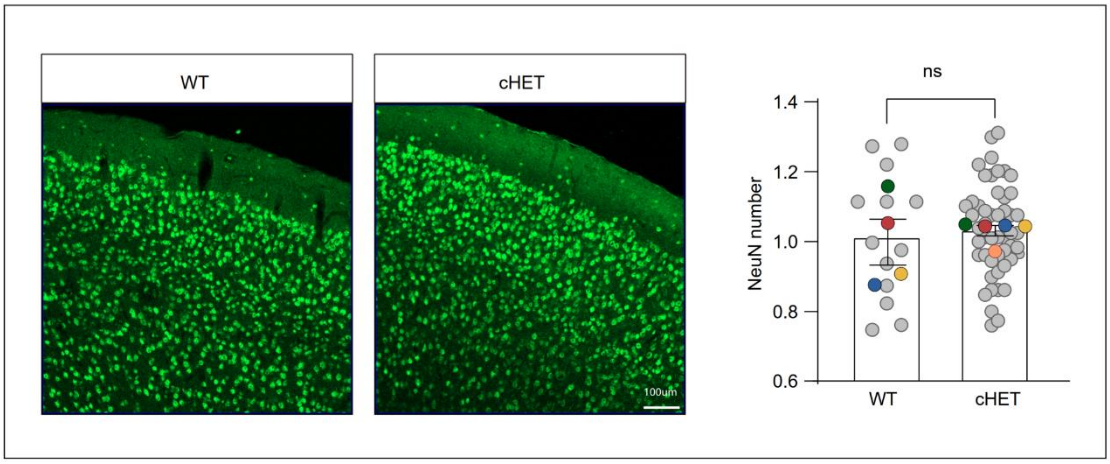


**Figure S12 Immunofluorescence Imaging and Quantitative Analysis of NeuN+ Neurons in WT and *Ints6* cHET Mice.** Left panels display NeuN immunofluorescence staining of cortical neurons in two-month-old WT and *Ints6* cHET mice, showing similar neuronal density. The right panel presents a statistical analysis of NeuN-positive cell counts across both genotypes, confirming no significant difference (ns) in the number of cortical neurons. Data are mean ± SEM. *P* values were determined from a 2-tailed unpaired Mann-Whitney test. ns = not significant. Each biological replicate (mouse) is color-coded; gray dots show individual data point, and colored dots indicate the mean per mouse.

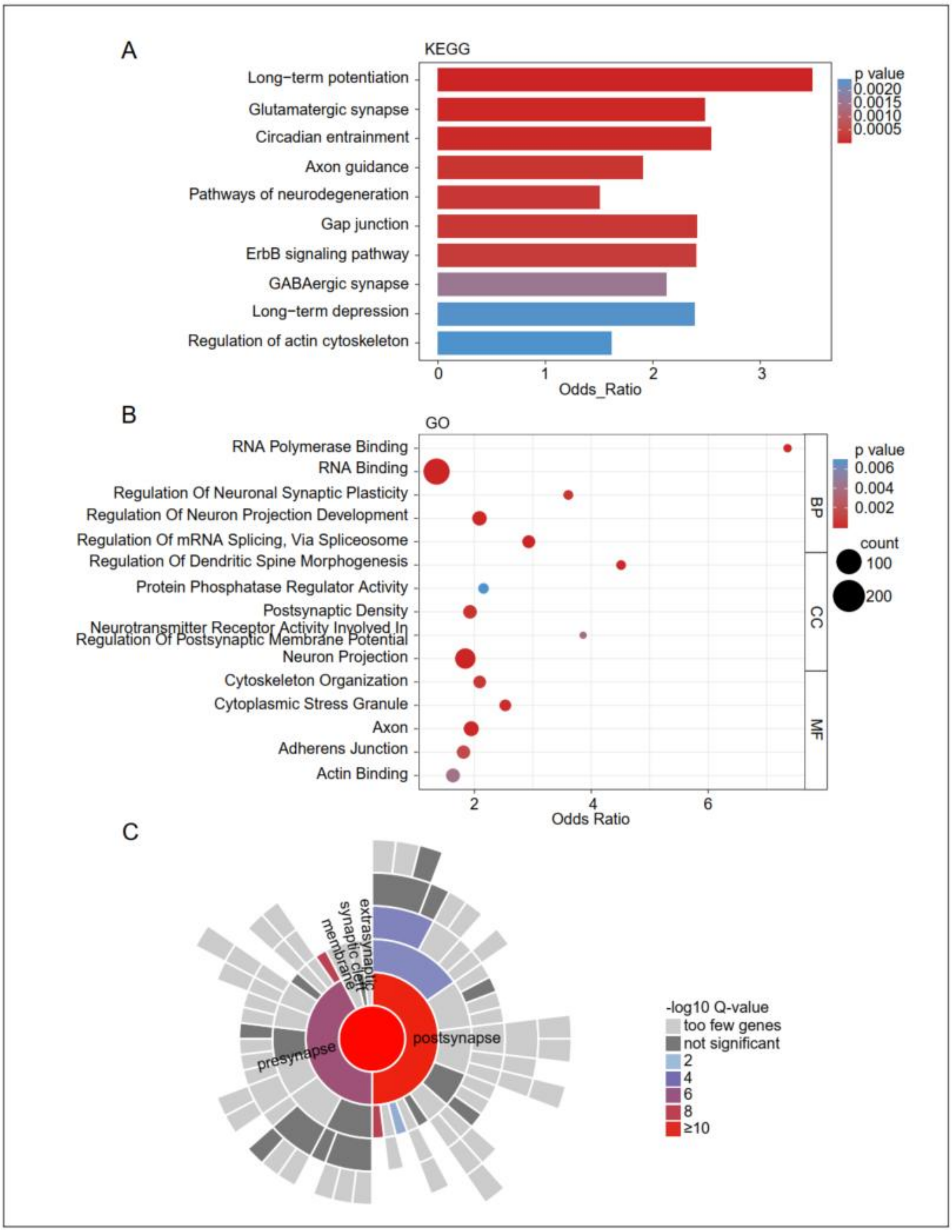


**Figure S13 KEGG and GO Enrichment Analysis of Differentially Expressed Genes in Cortical Tissues of Wild-Type and cHET Mice**. (**A**) Displays the enrichment of KEGG pathways in cortical tissues from two-month-old wild-type and heterozygous (cHET) mice. Bars represent the odds ratios for pathways with significantly altered expression. The color gradient from blue to red corresponds to increasing statistical significance from *P*<0.05 to *P*<0.0001. (**B**) Shows Gene Ontology enrichment for differentially expressed genes, focusing on neuronal functions and processes. Odds ratios are shown with a color coding that indicates the level of statistical significance. (**C**) The proteomic of isolated synapsins from WT and *Ints6* cHET mice and SynGO enrichment for differentially expressed proteins.

# Supplementary Data Methods

## Plasmid construction

The pCMV-INTS6 construct containing the full-length human INTS6 sequence (NM_012141.2) was purchased from Origene. This vector includes the 2661 bp coding region of INTS6, excluding the stop codon. Using pCMV6-INTS6 as a template, the entire INTS6 fragment was amplified and subsequently cloned into the pCAGGS-GFP vector using the AscI/XhoI restriction enzymes. To facilitate detection and protein expression studies, an HA tag (TACCCCTATGACGTCCCAGACTACGCA) was inserted between the full-length INTS6 sequence and the termination codon, resulting in the pCAGGS-hINTS6-HA-IRES-GFP wild-type (WT) plasmid. For the generation of mutant constructs, primers specific to the desired mutations were designed according to the positions of the mutations within the INTS6 coding sequence. The WT plasmid was used as a template for site-directed mutagenesis. The plasmids for each mutant were constructed by PCR amplification and verified for the presence of the mutations.

## Cell lines and culture conditions

Human embryonic kidney 293T cells (HEK293T, ATCC, CRL-3216™) were cultured at 37 °C in a humidified incubator with 5% $CO_2$ in Dulbecco's Modified Eagle's Medium (DMEM; Thermo Fisher Scientific, C11995500BT) supplemented with 10% fetal bovine serum (FBS; Sigma Aldrich, 341506). Cells were maintained in standard cell culture dishes (100 mm or 60 mm) and passaged at 70–90% confluence using 0.05% trypsin-EDTA, with routine propagation between passages.

## Western blot

Transfected cells were lysed with 2% SDS lysis buffer (2% SDS, 50mM pH 8 Tris-HCl,10mM EDTA,10% Glycerol) with protease inhibitor and phosphatase inhibitor. Proteins were denatured by heating at 95°C for 10 minutes. Protein concentration was quantified using the Thermo BCA Protein Assay Kit. Equal amounts of protein (10–20 μg) were separated by SDS-PAGE and transferred onto a PVDF membrane (Merck Millipore, IPVH00010). The membrane was blocked with 5% skim milk (Sigma Aldrich, A2058) at room temperature for 1 hour, then incubated overnight with primary

antibodies at 4°C. The following primary antibodies were used: HA (Cell Signaling Technology, 3724S), RNA Polymerase II, (Millipore, 05-623), Phospho RNA Polymerase II (S2) (Bethy, A300-654A), INTS6 (Santa Cruz, sc-376524), GFP (Invitrogen, A11122), and β-actin (Proteintech, 66009-1). The membrane was incubated with HRP-conjugated secondary antibody (Jackson ImmunoResearch) to detect primary antibody binding, and protein expression was quantified by density analysis using NIH ImageJ software.

**Immunofluorescence**

Embryonic mouse brain tissue was fixed in 4% paraformaldehyde (PFA) for 24 hours. For adult mice, brains were perfused with pre-chilled PBS, then fixed in 4% PFA for 24 hours. After fixation, the tissue was dehydrated in 15% and 30% sucrose, embedded in OCT, and stored at -80°C for quick freezing. Brain sections were cut at 20 μm for fetal tissue and 30 μm for adult tissue using a cryostat. Before staining, the sections were dried at 60°C and subjected to antigen retrieval as needed. The sections were washed with PBS and blocked for 1 hour at room temperature in PBS with 5% BSA and 0.3% Triton X-100. Primary antibodies were diluted in the same solution and incubated overnight at 4°C. The following primary antibodies were used: SATB2 (Abcam, ab51502), CTIP2 (Abcam, ab18465), TBR1 (Abcam, ab183032), Ki-67 (Cell Signaling Technology, 9129S), PAX6 (Abcam, ab195045), TBR2 (Invitrogen, 14-4875-82), Cleaved Caspase-3 (Cell Signaling Technology, 9661S), PSD95 (Invitrogen, 516900), and Synaptophysin (Sigma-Aldrich, S5768). After incubation, sections were washed six times with PBS containing 0.2% Triton X-100. Then, the sections were incubated with secondary antibodies (Jackson ImmunoResearch) diluted in PBS with 5% BSA and 0.3% Triton X-100 for 1 hour at room temperature, followed by washing. If needed, EdU (Thermo scientific, c10337) was stained before DAPI (Sigma-Aldrich, D9542) for nuclear staining. Finally, the sections were mounted with Fluoromount-G (SouthernBiotech, 0100-01). Due to the morphological anomalies of the mutant cortices, thalamic landmarks were used to match sections of different genotypes (1).

**In utero electroporation**

Pregnant C57BL/6J or *Inst6* $^{flox/+,\ cre/+}$ mice (E13-E16) were anesthetized with isoflurane and the abdomen was incised to expose the fetuses. A 1 mL syringe connected to a glass electrode was used to inject the plasmid mixture into the lateral

ventricle. After injection, the electrode was removed, and electroporation was performed using ECM 830 square wave electroporation system (BTX, 45-0662), delivering three 30V pulses with the positive electrode on the cortex and the negative electrode on the opposite side. During the electroporation, continuously replenish preheated physiological saline (37 °C) into the abdominal cavity.

**Mouse Golgi staining**

Golgi staining was performed using the FD Rapid Golgi Stain™ Kit (FD NeuroTechnologies, PK401). Briefly, after anesthesia with isoflurane, the mice were euthanized by cervical dislocation, and the brain was removed. The tissue was washed with $H_2O$ and immersed in a prepared soaking solution. The brain was soaked for 24 hours at room temperature in the dark, with the solution replaced the following day. The tissue was then transferred to Solution C and soaked for up to one week, replacing the solution after 24 hours. After soaking, the tissue was briefly dried and embedded in $H_2O$. The brain was sliced into 100 μm thick sections using a cryostat and mounted on gelatin-coated glass slides (1% gelatin, 0.05% potassium chromate). The sections were air-dried for two days, washed twice with $H_2O$, and then treated with an alkaline mixture for alkalization. After alkalization, the slices were washed twice with $H_2O$ and dehydrated sequentially in 50%, 75%, 95%, and 100% ethanol. Following dehydration, the slices were cleared in xylene for three times, mounted with resin, and air-dried in the dark. Finally, images were captured using microscope (Nikon, ECLIPSE Ti).

**Synaptosome Enrichment and DIA Mass Spectrometry**

Mouse brain tissues were homogenized on ice in Syn-PER™ Synaptic Protein Extraction Reagent (Thermo Fisher Scientific) supplemented with protease and phosphatase inhibitors. After sequential centrifugation at low and high speeds at 4°C, the synaptosome pellet was collected and resuspended for downstream analysis. Protein concentration was determined by BCA assay. Equal amounts of protein were reduced, alkylated, and digested with trypsin overnight at 37°C. Resulting peptides were desalted, dried, and reconstituted for data-independent acquisition (DIA) mass spectrometry analysis using a high-resolution mass spectrometer coupled with liquid chromatography.

**Cryo-EM sample preparation**

Mice were anesthetized, perfused with saline, and fixed with 4% PFA. After brain

extraction, hippocampal or cortical tissue was dissected, rinsed in 0.1M phosphate buffer (PB), and fixed overnight in 2.5% glutaraldehyde at 4°C. The tissue was post-fixed in 1% osmium tetroxide for 2 hours, then dehydrated through a graded ethanol series (30-100%) and 100% acetone. The tissue was permeated with a 1:1 mixture of acetone and embedding solution, then placed in pure embedding solution overnight. After embedding in 812 resin, the tissue was polymerized at 60°C for 48 hours. Ultra-thin sections (70 nm) were cut using a Leica UC7 slicer, stained with uranyl acetate and lead citrate, and imaged using transmission electron microscopy.

**Real-time Quantitative PCR (qPCR)**

RNA was extracted from tissues or cells using phenol-chloroform and reverse transcribed into cDNA with the RevertAid First Strand cDNA Synthesis Kit (Thermo scientific, K1622). For qPCR, 5 μL of 2× ChamQ Universal SYBR qPCR Master Mix (Vazyme, Q711-02), 0.2 μL of Primer F (10 μM), 0.2 μL of Primer R (10 μM), 1 μL of cDNA, and 3.6 μL of ddH2O were added. The reaction was run on a Bio-Rad CFX96 real-time PCR system with the following program: 5 minutes of initial denaturation at 95°C, 39 cycles of 10 seconds denaturation at 95°C, and 30 seconds of annealing/extension at 60°C.

**Mouse behavioral testing**

Three chamber social test: Place the mouse in the center chamber for 10 minutes to adapt. Then introduce an unfamiliar mouse (stranger 1) to one chamber and an object to the other, recording interactions for 10 minutes. Replace the object with another unfamiliar mouse (stranger 2) and record interactions with both mice for another 10 minutes. This method evaluates social preference by comparing responses to live mice versus an object.

Restricted and repetitive behaviors, RRBs: Place mice in a new cage to adapt for 6 minutes. Then, after this adaptation period, record their free activities for 6 minutes. Analyze behaviors such as grooming, digging, standing, and jumping using video software, which helps quantify the spontaneous repetitive activities characteristic of ASD.

Marble Burying Test: Place padding in a cage and arrange glass beads on top at fixed intervals. The beads serve as unfamiliar and potentially threatening objects. The instinctual behavior of mice burying these beads reflects anxiety-like reactions. Record

the number of beads buried more than 50% by the mice within a predetermined time to analyze their anxiety levels. This method quantifies instinctive anxiety responses in experimental mice.

Open field test: In the open field test, the arena is divided into a 16x16 grid, with the central 4x4 squares designated as the center area. Record the total movement distance of the mice over 10 minutes and the time they spend exploring the central area. This test evaluates the mice's general activity levels and anxiety-related behavior based on their willingness to explore a novel, open space.

Light dark test: This test uses a setup divided into a bright and a dark box to measure mouse anxiety by balancing their curiosity and fear of light. Record the time and distance mice spend in the bright box over 10 minutes to assess their anxiety levels based on their willingness to explore despite their innate fear of light.

Elevated plus maze: The elevated plus maze evaluates mouse anxiety by leveraging their curiosity to explore open arms and their natural preference for darkness in closed arms. This setup creates a conflict between exploratory behavior and avoidance, indicative of anxiety. Record the time and distance mice spend in both the open and closed arms over a 6-minute period to assess their anxiety-related behaviors.

Y-maze: The Y-maze test evaluates rodents' spatial recognition and memory by observing their exploration. The maze has three arms labeled A, B, and C. An "effective behavior" is noted when a mouse sequentially explores all three arms without repetition. Record the proportion of effective behaviors over an 8 minutes period to assess cognitive abilities.

Morris water maze: Before the experiment, mice acclimate to handlers and undergo preliminary swimming tests, excluding those with movement disorders. In the test, mice start from four random points facing the pool wall to find a hidden platform, recording time (escape latency) and distance traveled. If a mouse doesn't find the platform within 60 seconds, it's guided there and stays for 10 seconds. On the sixth day, without the platform, record the number of platform crossings and the time and distance spent in the former platform's quadrant within 60 seconds.

**RNA sequencing**

Tissue or cell samples were lysed with TRIzol (Thermo scientific, 15596026), and total

RNA was extracted using phenol-chloroform extraction. RNA concentration was measured with a Qubit, and 1 μg was used for library construction. Libraries were sequenced on the Illumina NovaSeq 6000 platform using a 2 x 150 paired-end configuration. Sequencing images were converted to raw sequence data (reads) using CASAVA base calling. Following quality control, reads were aligned to the reference genome using STAR software. Transcript assembly was performed with StringTie (v1.3.1c), which also quantified read counts for known genes. Differential expression analysis was conducted with DESeq2.

**RNA immunoprecipitation sequencing (RIP-Seq)**

The RIP experiment was conducted following the manufacturer's protocol (Guangzhou Saicheng Biotechnology, KT102). Briefly, animal tissue was homogenized using a Dounce homogenizer, and individual cells were separated by passing through a 0.45 μm cell strainer for cell counting, requiring approximately 1–2 × $10^7$ cells. Cells were lysed in pre-chilled cell lysis buffer at 4°C for 1–2 hours. After lysis, the supernatant was carefully removed and replaced with RIP buffer. RNAse inhibitors, pre-washed magnetic beads, and RNA Polymerase II (Millipore, 05-623) or IgG were added, and the mixture was incubated overnight at 4°C with gentle agitation. Ten percent of the sample was reserved as "input" and stored at -80°C. The following day, the bound magnetic beads were washed with RIP buffer, and RNA was extracted using a phenol-chloroform extraction protocol. The purified RNA was stored at -80°C until further processing. For sequencing, RNA samples were submitted to Xuran Biotechnology for library construction, followed by high-throughput sequencing on the Illumina platform using paired-end sequencing.

**CUT & Tag**

The CUT&Tag experiment was performed following the protocol provided by the Hieff NGS® G-Type In-Situ DNA Binding Profiling Library Prep Kit for Illumina (YEASEN, 12598ES). Briefly, activated ConA-coated magnetic beads were added to resuspended cells and incubated at room temperature. RNA Polymerase II (Millipore, 05-623), secondary antibody, and highly active pA-Tn5 transposase were then added to the ConA bead-bound cells and incubated at room temperature for 1 hour. After removing non-specifically bound material, transposase was activated by adding an activation buffer to cleave DNA fragments associated with the target protein. The cleaved genomic DNA

was harvested, followed by library amplification and purification using N5 and N7 adapters (YEASEN, 12416ES). The final library was submitted to Xuran Biotechnology for high-throughput sequencing on the Illumina platform using paired-end sequencing.

**Sequencing data analysis**

After performing quality control on the raw sequencing data, the preprocessed reads were aligned to the reference genome of the target species using the STAR (2.5.2b) aligner. Peak calling was carried out for each sample using Model-based Analysis for ChIP-Seq (MACS2), and the identified peaks were subsequently annotated to the nearest genes. Overlap analysis of peak regions was performed using a web-based tool (https://jvenn.toulouse.inrae.fr/app/index.html), and downstream functional enrichment analysis was conducted using Enrichr (https://maayanlab.cloud/Enrichr/) and SynGO (https://www.syngoportal.org/). Visualization of the results, including bubble and bar plots, was performed with the ggplot2 package in R (3.6.1).

**Key resources table**

| Antibodies | | |
|---|---|---|
| Anti-RNA polymerase II Antibody, clone CTD4H8 | Millipore | 05-623 |
| Rabbit anti-Phospho RNA Polymerase II (S2) Antibody | Bethy | A300-654A |
| DICE1 Antibody (H-6) | Santa Cruz | sc-376524 |
| Anti-SATB1 + SATB2 antibody [SATBA4B10] - C-terminal | Abcam | ab51502 |
| Anti-Ctip2 antibody [25B6] | Abcam | ab18465 |
| Anti-TBR1 antibody [EPR8138(2)] | Abcam | Ab183032 |
| CoraLite®594-conjugated TBR1 Monoclonal antibody | Proteintech | CL594-66564 |
| EOMES Monoclonal Antibody (Dan11mag) | Invitrogen | 14-4875-82 |
| Anti-PAX6 antibody [EPR15858] | Abcam | ab195045 |
| Ki-67 (D3B5) Rabbit mAb | Cell Signaling Technology | 9129S |

| | | |
|---|---|---|
| Cleaved Caspase-3 (Asp175) Antibody | Cell Signaling Technology | 9661S |
| GFP Polyclonal Antibody | Invitrogen | A11122 |
| HA-Tag (C29F4) Rabbit mAb | Cell Signaling Technology | 3724S |
| PSD-95 Polyclonal Antibody | Invitrogen | 516900 |
| Monoclonal Anti-Synaptophysin antibody produced in mouse | Sigma-Aldrich | S5768 |
| Beta Actin Monoclonal antibodies (2D4H5) | Proteintech | 66009-1 |
| Cy™3 AffiniPure™ Goat Anti-Rabbit IgG (H+L) | Jackson ImmunoResearch | 111-165-003 |
| Cy™3 AffiniPure™ Goat Anti-Mouse IgG (H+L) | Jackson ImmunoResearch | 115-165-003 |
| Alexa Fluor® 488 AffiniPure™ Goat Anti-Mouse IgG, light chain specific | Jackson ImmunoResearch | 115-545-174 |
| Alexa Fluor® 488 AffiniPure™ Goat Anti-Rabbit IgG (H+L) | Jackson ImmunoResearch | 111-545-144 |

| Chemicals and Kit | | |
|---|---|---|
| DAPI | Sigma-Aldrich | D9542 |
| Paraformal delyde | Sigma-Aldrich | 158127-500G |
| BSA | Sigma-Aldrich | A1933 |
| Fluoromount-G | SouthernBiotech | 0100-01 |
| Click-iT™ EdU Cell Proliferation Kit for Imaging, Alexa Fluor™ 488 dye | Thermo scientific | c10337 |
| FD Rapid GolgiStain™ Kit | FD Neuro Technologies | PK401 |
| SDS | Sigma-Aldrich | 74255 |
| Tris | VWR | 0497-5KG |
| Glycine | VWR | 0167-5KG |
| 2-Mercaptoethanol | Sigma-Aldrich | M3148 |
| Bromophenol blue | Diamond | A100449 |
| TRIzol | Thermo Fisher | 15596026 |
| RevertAid First Strand cDNA Synthesis Kit | Thermo Fisher | K1622 |

| | | |
|---|---|---|
| ChamQ Universal SYBR qPCR Master Mix | Vazyme | Q711-02 |
| FastDigest SgsI | Thermo Fisher | FD1894 |
| FastDigest XhoI | Thermo Fisher | FD0694 |
| DMEM | Gibco$^{TM}$ | C11995500BT |
| Neofect™ DNA transfection reagent | NEOFECT | TF201201 |
| neurobasal | invitrogen | 21103 |
| B27 | Gibco$^{TM}$ | 17504-044 |
| Penicillin-Streptomycin | Gibco$^{TM}$ | 11360070 |
| GlutaMAX™ Supplement | Gibco$^{TM}$ | 35050061 |
| HBSS, no calcium, no magnesium, no phenol red | Gibco$^{TM}$ | 14175095 |
| Poly-D-Lysine | Gibco$^{TM}$ | A3890401 |
| MEM | Gibco$^{TM}$ | 11095-072 |
| DPBS | Gibco$^{TM}$ | 14190144 |
| papain | worthington | 9001-73-4 |

| Oligonucleotides and Recombinant DNA | |
|---|---|
| CAGGS-HA-INTS6(WT) | GgcgcgccATGCCCATCTTACTG<br>GctcgagTTAtgcgtagtctgggacgtc |
| CAGGS-HA-INTS6(S91F) | CTTACGACTCTTGGCCAATTCCTAAGGACAGCTTTTG<br>CAAAAGCTGTCCTTAGGAATTGGCCAAGAGTCGTAAG |
| CAGGS-HA-INTS6(Y111C) | GGCATAGACAACTGTGGGCAGGGAAGAAACCC<br>GGGTTTCTTCCCTGCCCACAGTTGTCTATGCC |
| CAGGS-HA-INTS6(T137I) | GGAGCAAGTTGACTACCATCAGTGGAGTCCAGGATGAG;<br>CTCATCCTGGACTCCACTGATGGTAGTCAACTTGCTCC |
| CAGGS-HA-INTS6(R206C) | GTGAAGTGACAGGCGGCTGTTCATATTCTGTGTGTTC<br>GAACACACAGAATATGAACAGCCGCCTGTCACTTCAC |
| CAGGS-HA-INTS6(V210M) | CGGCCGTTCATATTCTATGTGTTCTCCAAGAATGCTT<br>AAGCATTCTTGGAGAACACATAGAATATGAACGGCCG |
| CAGGS-HA-INTS6(Q228E) | GTCCTTGGTGCAGAAAGTAGAAAGTGGGGTGG<br>CCACCCCACTTTCTACTTTCTGCACCAAGGAC |

| | |
|---|---|
| CAGGS-HA-INTS6(P284S) | GGTTCCTATAGGTCATTGGTCTGTTCCAGAGTC<br>GACTCTGGAACAGGCCAATGACCTATAGGAACC |
| CAGGS-HA-INTS6(H400R) | GATGACTTGTTTAAAGTGCGTAAAGCAAAACC<br>GGTTTTGCTTTAcGCACTTTAAACAAGTCATC |
| CAGGS-HA-INTS6(F35Hfs*41) | AAGGCGCGGTAGAGACCCATGAAGCTCCGTGCCC<br>GG<br>CCGGGCACGGAGCTTCATGGGTCTCTACCGCGCC<br>TT |
| CAGGS-HA-INTS6(Q185*) | CTAtgcgtagtctgggacgtcataggggtaTTCTGATTCTACTGA<br>CATG |
| CAGGS-HA-INTS6(V278Sfs*37) | CCAAATCCTAAAACTGGGTCCTATAGGTCATTGG<br>CC<br>GGCCAATGACCTATAGGACCCAGTTTTAGGATTT<br>GG |
| CAGGS-HA-INTS6(P323Lfs*12) | TCAtgcgtagtctgggacgtcataggggtaGTGGTGAAGGTTCC<br>AACTCATATTTGTCAAAAGAAGTTTATCAATAAC<br>CAT |
| CAGGS-HA-INTS6(K326*) | TTAtgcgtagtctgggacgtcataggggtaGTCAAAAGGAAGTT<br>TATC |
| CAGGS-HA-INTS6(W408*) | CTAtgcgtagtctgggacgtcataggggtaCTTCAATGTTGGTTT<br>TGCT |
| CAGGS-HA-INTS6(L536*) | CTAtgcgtagtctgggacgtcataggggtaATCCTTATTCAGCAA<br>AGC |
| CAGGS-HA-INTS6(E603Tfs*2) | TCAtgcgtagtctgggacgtcataggggtaAGTTCTTAGTGGAG<br>AAGGTAC |
| CAGGS-HA-INTS6(R610*) | TTAtgcgtagtctgggacgtcataggggtaTGGCTGATCAGGATC<br>AAGT |
| CAGGS-HA-INTS6(S737*) | TTAtgcgtagtctgggacgtcataggggtaAAATTCCGTATCCAT<br>AGCA |
| mINTS6 shRNA-1 | 1: GCCAATCAAATCAACCATATT<br>2: AATATGGTTGATTTGATTGGC |
| mINTS6 shRNA-2 | 1: ACTGACCTGTGTCAACTTATT<br>2: AATAAGTTGACACAGGTCAGT |

| Software and algorithms | |
|---|---|
| FastQC (v0.11.5) | http://www.bioinformatics.babraham.ac.uk/projects/fastqc |
| bwa (0.7.10) | http://bio-bwa.sourceforge.net |
| STAR (2.5.2b) | https://github.com/alexdobin/STAR |
| Samtools(1.3.1) | http://samtools.sourceforge.net |
| picard-tools (2.6.0) | https://broadinstitute.github.io/picard/ |

| | |
|---|---|
| MACS2 | https://github.com/macs3-project/MACS/wiki/Install-macs2 |
| homer (4.11-2) | http://homer.ucsd.edu/homer |
| R (3.6.1) | http://www.r-project.org/ |
| imaris | https://imaris.oxinst.com/ |
| imajeJ | https://imagej.nih.gov/ij/ |
| GraphPad Prism9 | https://www.graphpad.com/ |
| LAS X | https://www.leica-microsystems.com |
| ZEN blue | http://www.zeiss.com |

1. Orosco LA, Ross AP, Cates SL, Scott SE, Wu D, Sohn J, et al. Loss of Wdfy3 in mice alters cerebral cortical neurogenesis reflecting aspects of the autism pathology. *Nat Commun.* 2014;5:4692.
