# Supplementary material for: Disrupting integrator complex subunit INTS6 causes neurodevelopmental disorders and impairs neurogenesis and synapse development
Source: J Clin Invest. 2025 Sep 18;135(22):e191729. doi: 10.1172/JCI191729 (PMC12618080; doi:10.1172/JCI191729)
Supplement: Unedited blot and gel images [file jci-135-191729-s237.pdf]

Antibody: HA (Cell Signaling Technology, 3724S)

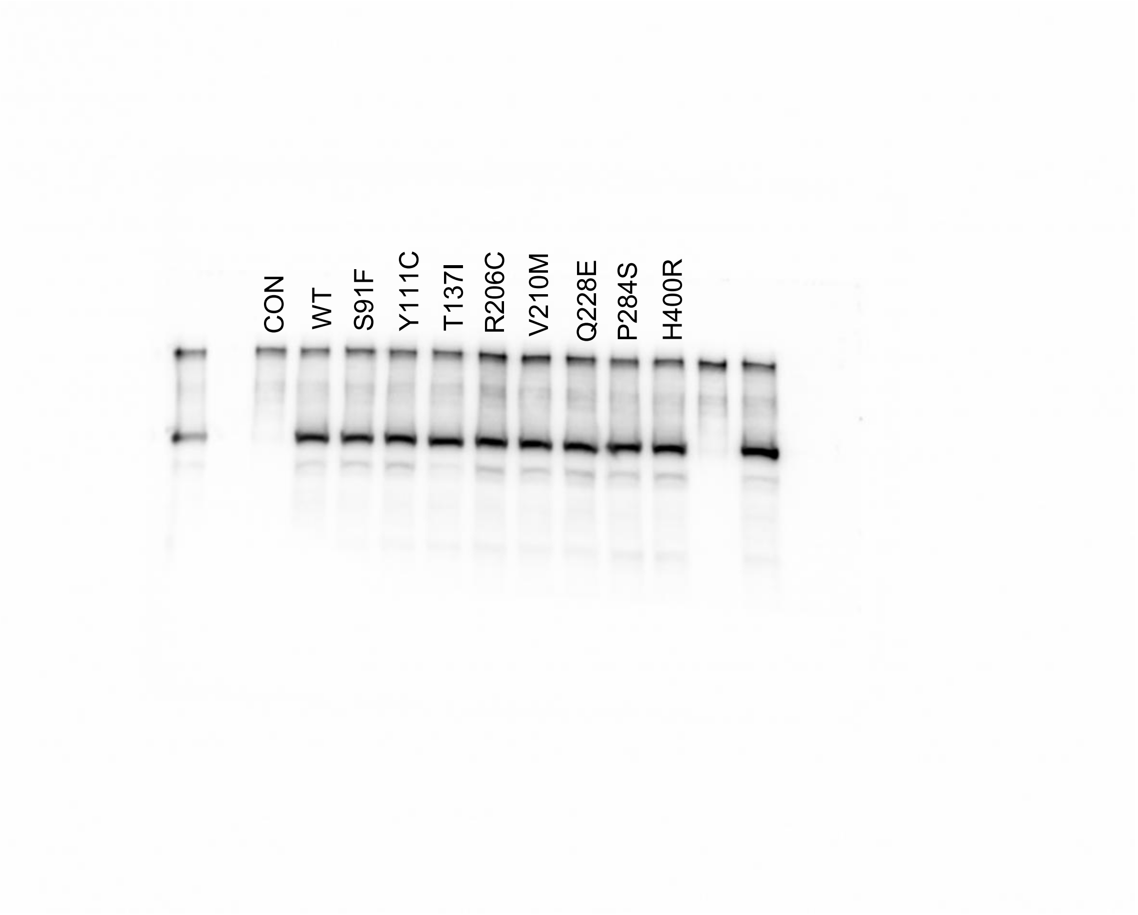

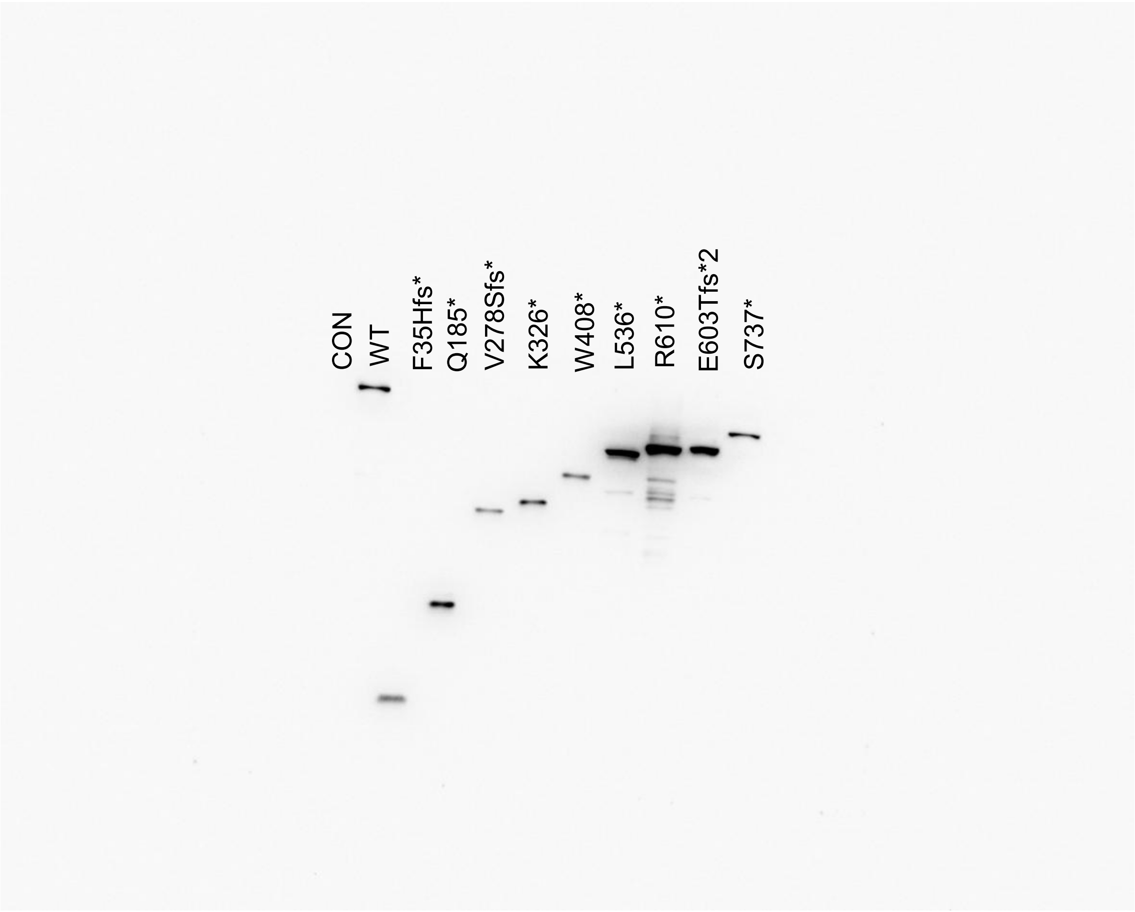

Figure S8H

Antibody: Phospho RNA Polymerase II (S2) (Bethy, A300-654A)

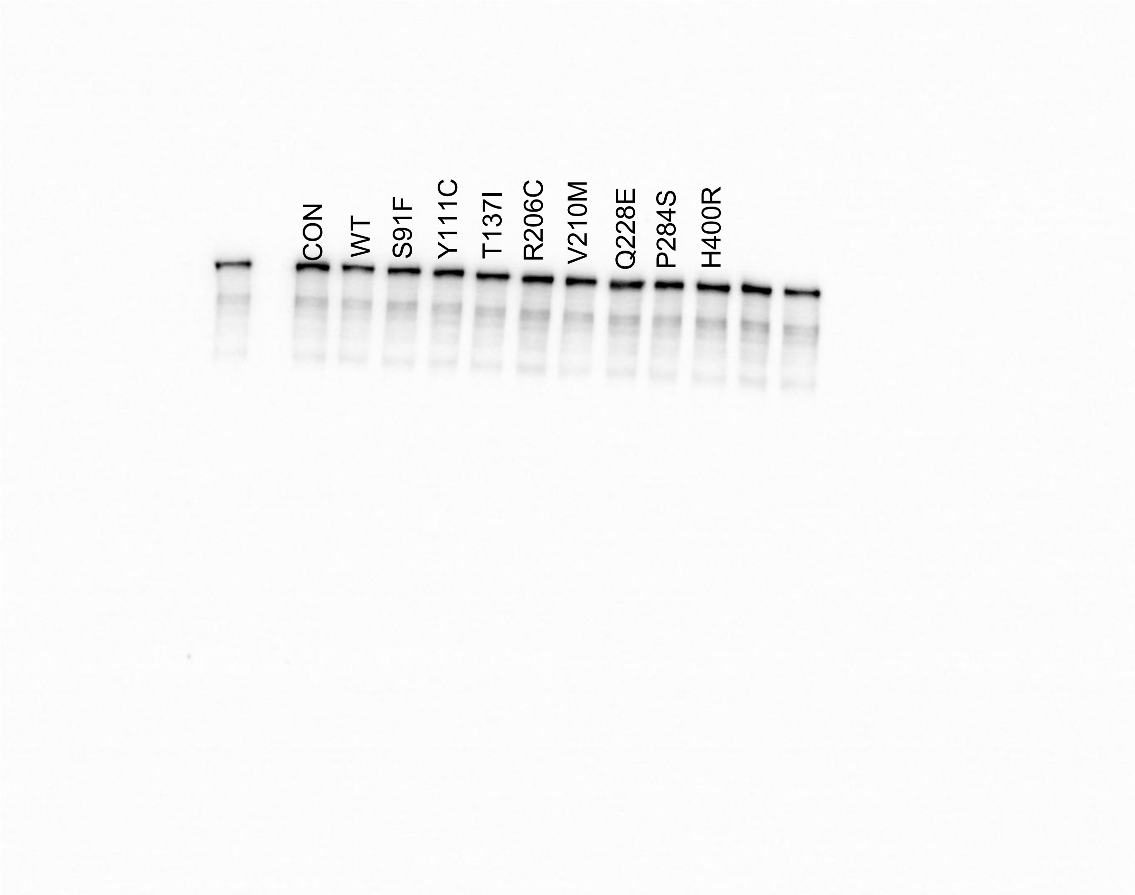

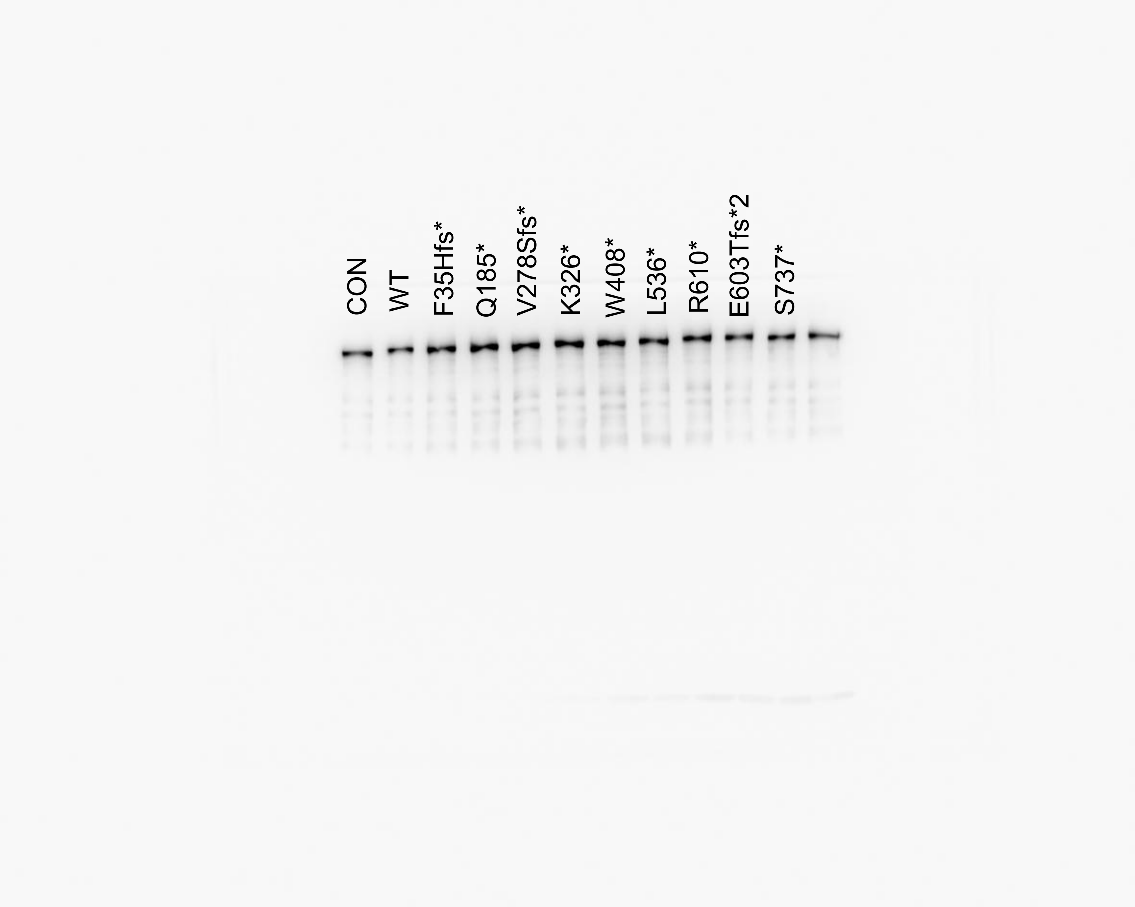

Figure S8H

Antibody: RNA Polymerase II, (Millipore, 05-623)

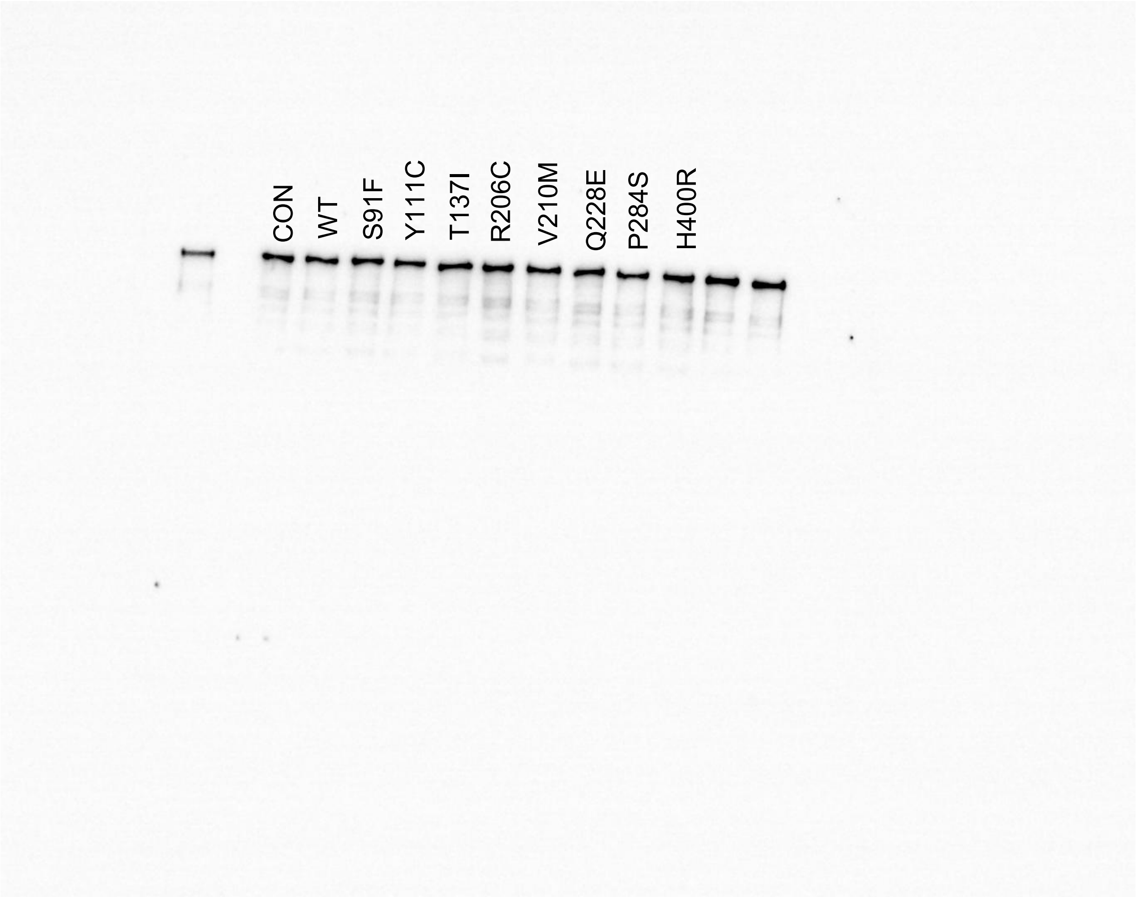

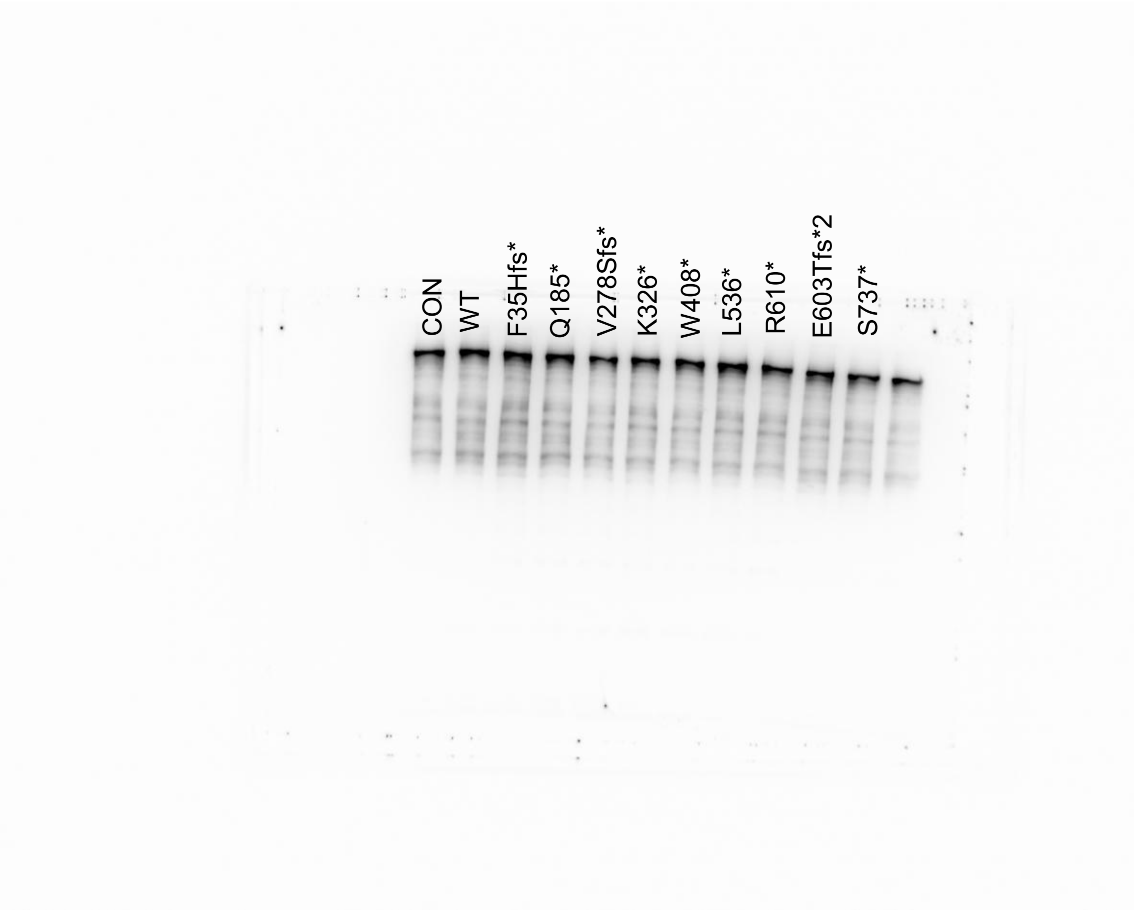

Figure S8H

Antibody: β-actin (Proteintech, 66009-1)

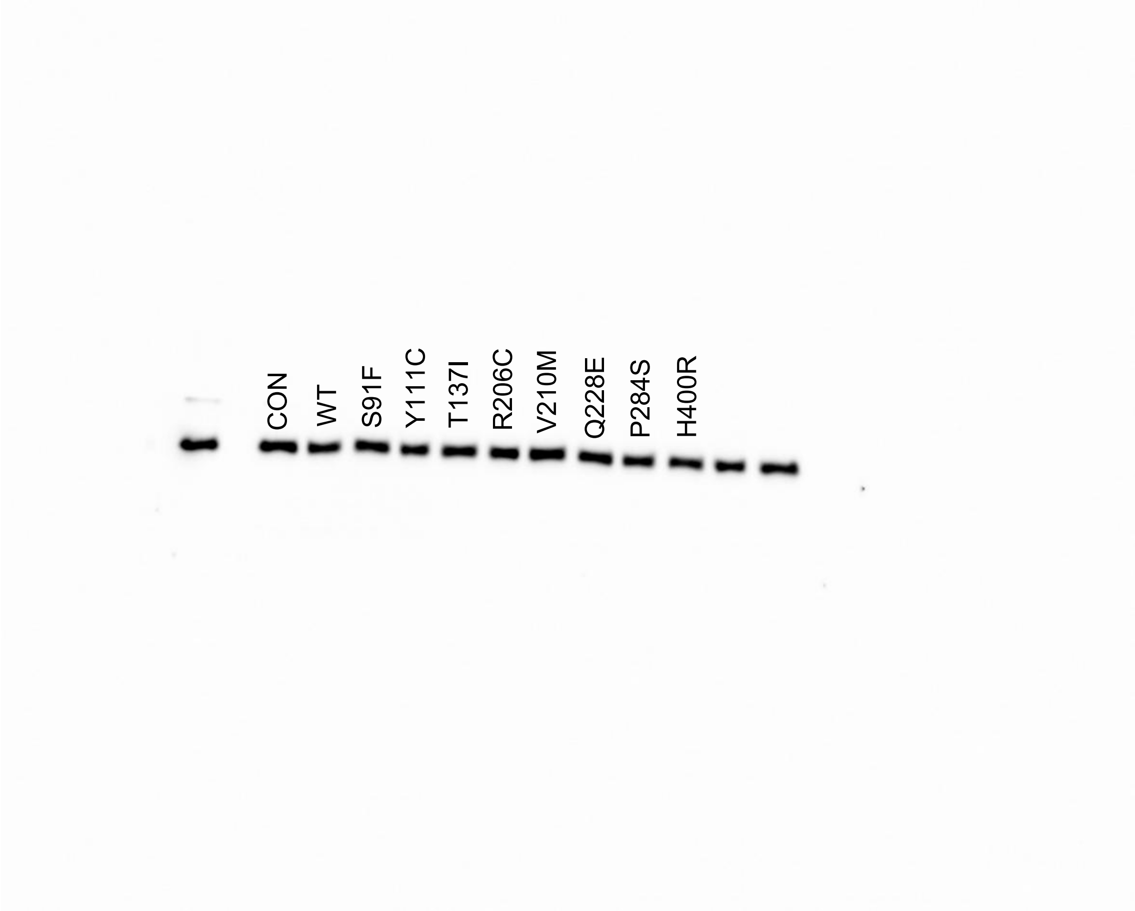

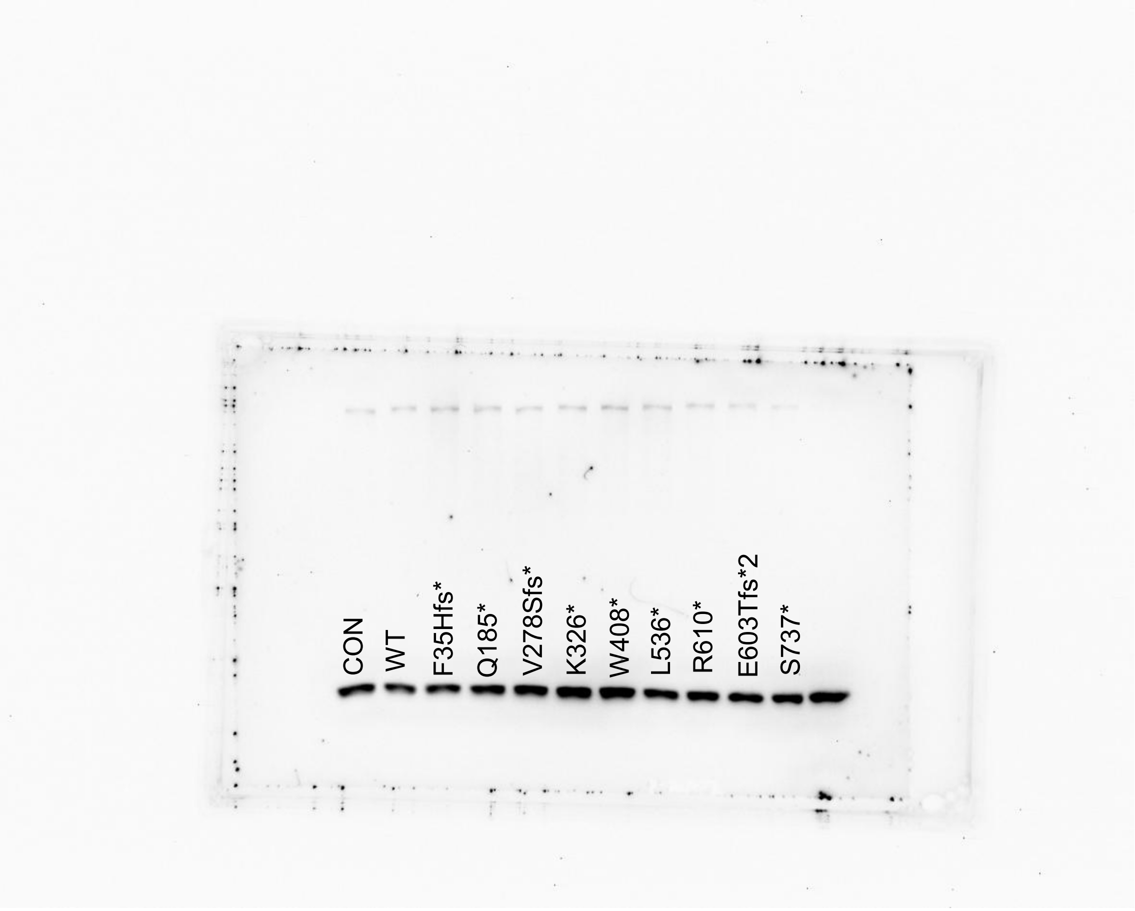

Figure S8H

Antibody: Phospho RNA Polymerase II (S2) (Bethy, A300-654A)

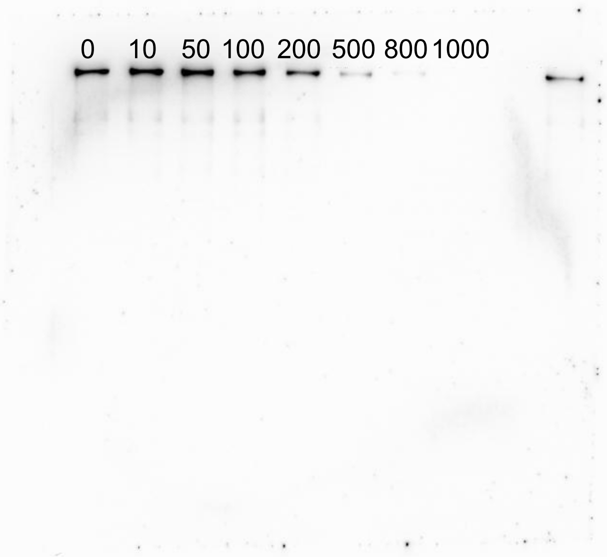

Figure S8I

Antibody: RNA Polymerase II, (Millipore, 05-623)

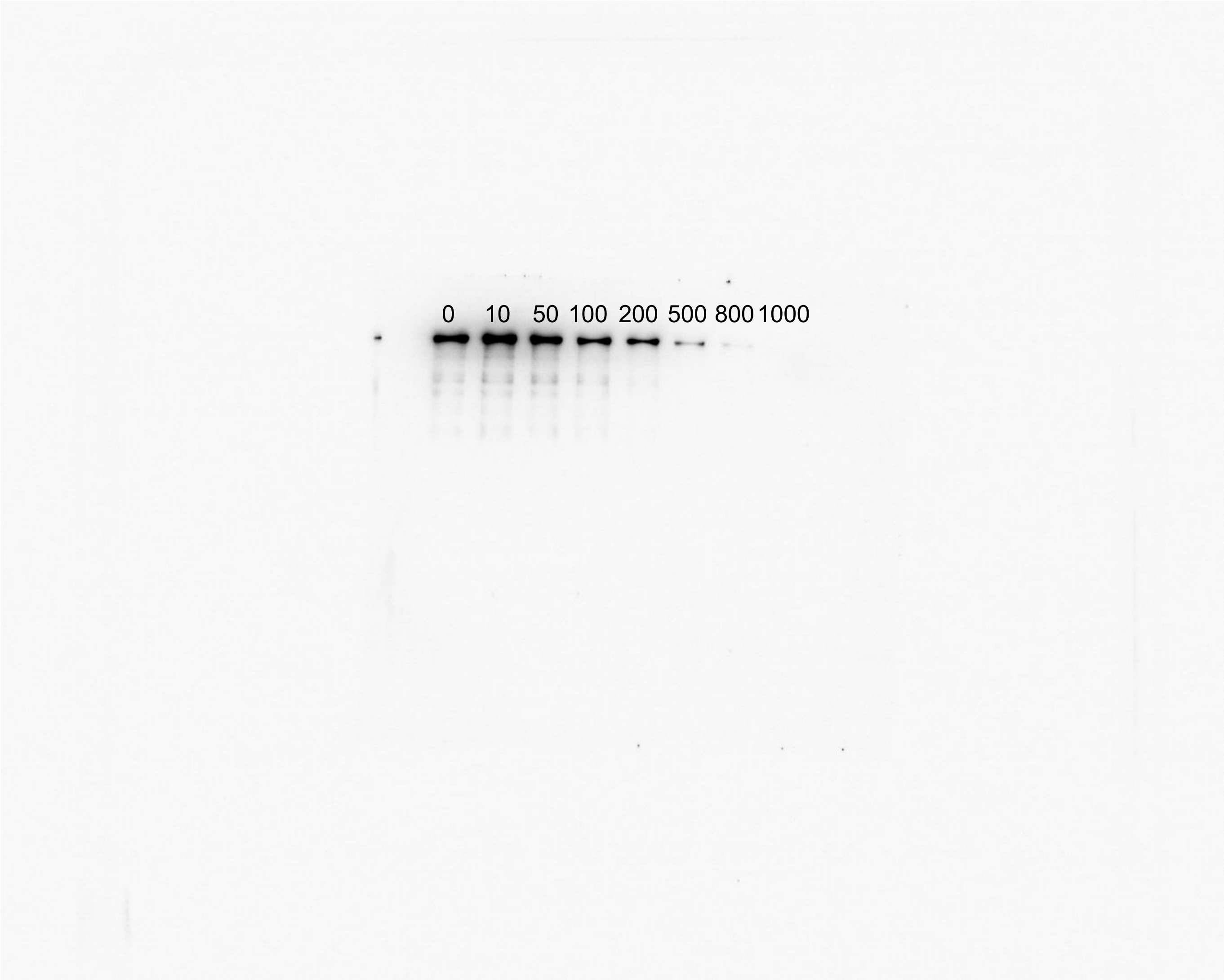

Figure S8I

Antibody: β-actin (Proteintech, 66009-1)

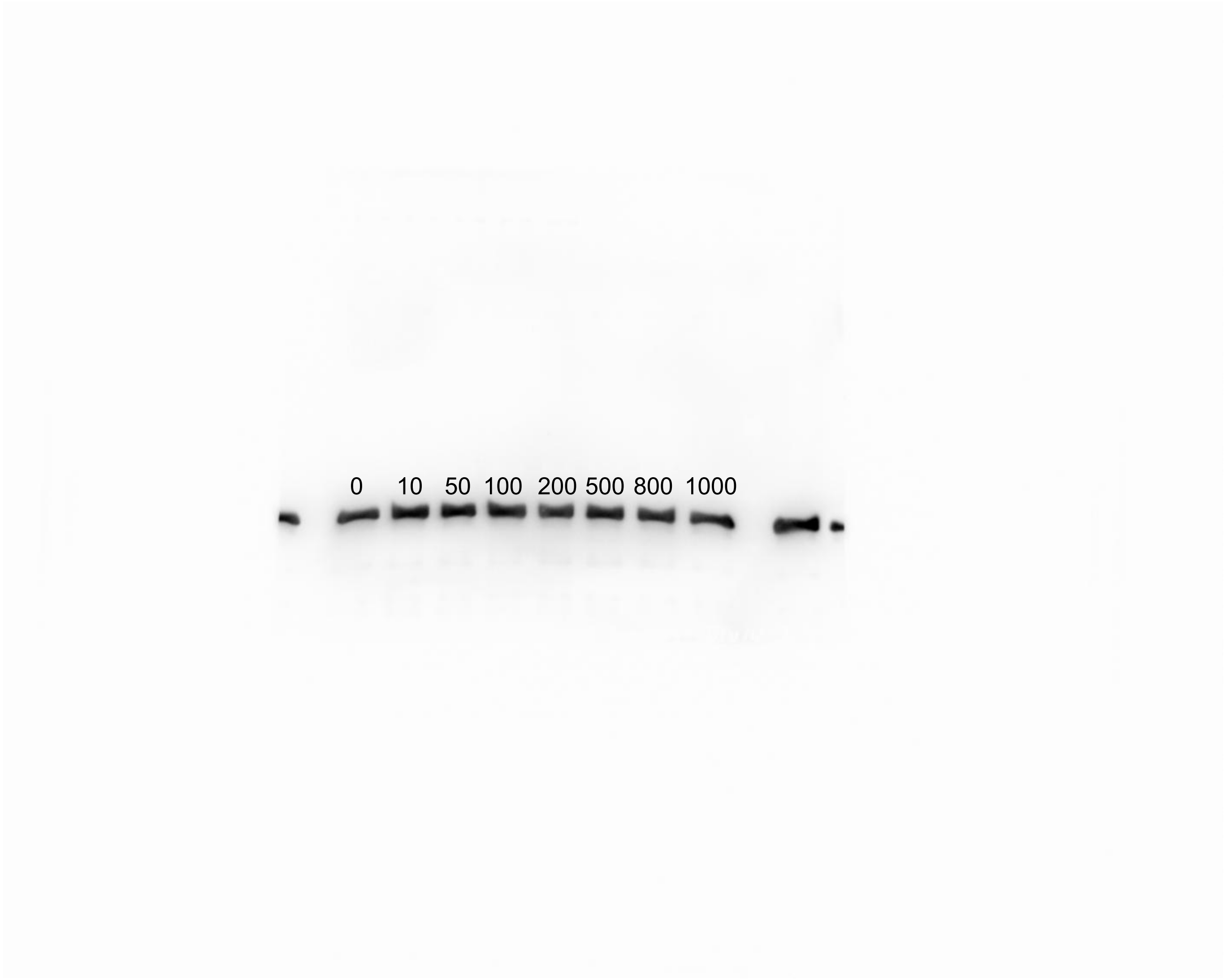

Figure S8I
